# A cluster of multidrug-resistant *Mycobacterium tuberculosis* among patients arriving in Europe from the Horn of Africa: a molecular epidemiological study

**DOI:** 10.1016/S1473-3099(18)30004-5

**Published:** 2018-04

**Authors:** Timothy M Walker, Matthias Merker, Astrid M Knoblauch, Peter Helbling, Otto D Schoch, Marieke J van der Werf, Katharina Kranzer, Lena Fiebig, Stefan Kröger, Walter Haas, Harald Hoffmann, Alexander Indra, Adrian Egli, Daniela M Cirillo, Jérôme Robert, Thomas R Rogers, Ramona Groenheit, Anne T Mengshoel, Vanessa Mathys, Marjo Haanperä, Dick van Soolingen, Stefan Niemann, Erik C Böttger, Peter M Keller, MDR-TB Cluster Consortium, Korkut Avsar, Christoph Bauer, Enos Bernasconi, Emanuele Borroni, Sergio Brusin, Mireia Coscollá Dévis, Derrick W. Crook, Martin Dedicoat, Margaret Fitzgibbon, Sébastien Gagneux, Francisca Geiger, Jean-Paul Guthmann, David Hendrickx, Sabine Hoffmann-Thiel, Jakko van Ingen, Sarah Jackson, Katia Jaton, Christoph Lange, Jessica Mazza Stalder, Joan O'Donnell, Onya Opota, Tim E.A. Peto, Benjamin Preiswerk, Emma Roycroft, Mariko Sato, Regina Schacher, Bettina Schulthess, E. Grace Smith, Hanna Soini, Wladimir Sougakoff, Elisa Tagliani, Christian Utpatel, Nicolas Veziris, Christiane Wagner-Wiening, Mark Witschi

**Affiliations:** a Nuffield Department of Medicine, University of Oxford, John Radcliffe Hospital, Oxford, UK; b Swiss Tropical and Public Health Institute, University of Basel, Basel, Switzerland; c Division of Communicable Diseases, Federal Office of Public Health, Bern, Switzerland; d University of Zurich and Tuberculosis Center of the Swiss Lung Association, Zurich, Switzerland; e European Centre for Disease Prevention and Control, Solna, Sweden; f National Tuberculosis Reference Laboratory, National Institute for Public Health and the Environment, Bilthoven, Netherlands; g Center for Infectious Disease Research, Diagnostics and Perinatal Screening, Center for Infectious Disease Research, Diagnostics and Perinatal Screening, Bilthoven, Netherlands; h Molecular and Experimental Mycobacteriology, Research Center Borstel, Borstel, Germany; i National Reference Center for Mycobacteria, Research Center Borstel, Borstel, Germany; j Respiratory Infections Unit, Department of Infectious Disease Epidemiology, Robert Koch Institute, Berlin, Germany; k SYNLAB Gauting and Institute of Microbiology and Laboratory Medicine, World Health Organization Supranational Reference Laboratory of Tuberculosis, Munich-Gauting, Germany; l Austrian Reference Laboratory for Mycobacteria, Austrian Agency for Health and Food Safety, Vienna, Austria; m Clinical Microbiology, University Hospital Basel, Basel, Switzerland; n Applied Microbiology Research, Department of Biomedicine, University of Basel, Basel, Switzerland; o Emerging Bacterial Pathogens Unit, Division of Immunology and Infectious Diseases, San Raffaele Scientific Institute, San Raffaele Hospital, Milan, Italy; p Assistance publique-Hôpitaux de Paris, Centre National de Référence de Mycobactéries et de la Résistance des Mycobactéries aux Antituberculeux, Bactériologie-Hygiène, Hôpitaux Universitaires Pitié Salpêtrière and Sorbonne Universités, Centre d'Imagerie Médicale Italie-Paris, CR7, Institut national de la santé et de la recherche médicale U1135, Paris, France; q Irish Mycobacteria Reference Laboratory, St James's Hospital Dublin, Ireland; r Department of Clinical Microbiology, Trinity College Dublin, Dublin, Ireland; s Department of Microbiology, Public Health Agency of Sweden, Solna, Sweden; t National Reference Laboratory for Mycobacteria, Norwegian Institute of Public Health, Oslo, Norway; u Program Tuberculosis and Mycobacteria, Operational Direction Communicable and Infectious Diseases, Scientific Institute of Public Health (Site Ukkel), Ukkel, Belgium; v National Institute for Health and Welfare, Department of Infectious Diseases Surveillance and Control, Turku, Finland; w Department of Health Security, National Institute for Health and Welfare, Helsinki, Finland; x Institute of Medical Microbiology, National Center for Mycobacteria, University of Zurich, Zurich, Switzerland

## Abstract

**Background:**

The risk of tuberculosis outbreaks among people fleeing hardship for refuge in Europe is heightened. We describe the cross-border European response to an outbreak of multidrug-resistant tuberculosis among patients from the Horn of Africa and Sudan.

**Methods:**

On April 29 and May 30, 2016, the Swiss and German National Mycobacterial Reference Laboratories independently triggered an outbreak investigation after four patients were diagnosed with multidrug-resistant tuberculosis. In this molecular epidemiological study, we prospectively defined outbreak cases with 24-locus mycobacterial interspersed repetitive unit-variable number tandem repeat (MIRU-VNTR) profiles; phenotypic resistance to isoniazid, rifampicin, ethambutol, pyrazinamide, and capreomycin; and corresponding drug resistance mutations. We whole-genome sequenced all *Mycobacterium tuberculosis* isolates and clustered them using a threshold of five single nucleotide polymorphisms (SNPs). We collated epidemiological data from host countries from the European Centre for Disease Prevention and Control.

**Findings:**

Between Feb 12, 2016, and April 19, 2017, 29 patients were diagnosed with multidrug-resistant tuberculosis in seven European countries. All originated from the Horn of Africa or Sudan, with all isolates two SNPs or fewer apart. 22 (76%) patients reported their travel routes, with clear spatiotemporal overlap between routes. We identified a further 29 MIRU-VNTR-linked cases from the Horn of Africa that predated the outbreak, but all were more than five SNPs from the outbreak. However all 58 isolates shared a capreomycin resistance-associated *tlyA* mutation.

**Interpretation:**

Our data suggest that source cases are linked to an *M tuberculosis* clone circulating in northern Somalia or Djibouti and that transmission probably occurred en route before arrival in Europe. We hypothesise that the shared mutation of *tlyA* is a drug resistance mutation and phylogenetic marker, the first of its kind in *M tuberculosis* sensu stricto.

**Funding:**

The Swiss Federal Office of Public Health, the University of Zurich, the Wellcome Trust, National Institute for Health Research (NIHR) Oxford Biomedical Research Centre (BRC), the Medical Research Council, BELTA-TBnet, the European Union, the German Center for Infection Research, and Leibniz Science Campus Evolutionary Medicine of the Lung (EvoLUNG).

## Introduction

WHO's 2017 global tuberculosis report^1^ contained the sobering news that the tuberculosis epidemic is larger than previously thought. Worse still, among an estimated 10·4 million new cases were 600 000 patients with rifampicin resistance or multidrug-resistant tuberculosis in 2016. Although the global burden of disease is unequally shared, population movement does lead to appearance of strains normally associated with one country or region in another country or region—a phenomenon historically linked to human migration, trade, and conquest.^2^ War, government oppression, and economic inequality are the modern day forces driving people to Europe from its southern borders.^3^ Climate change is likely to amplify these push factors in years to come.^4^ In 2015 alone, more than 1 000 000 people did the dangerous journey to Europe in search of sanctuary or a better life than before.^5^

A common route for refugees and migrants who make the journey from the Horn of Africa is through Libya where human traffickers detain their clients in overcrowded and unsanitary conditions. With a high prevalence of infectious diseases in their countries of origin, risk of transmission is heightened under these conditions, while access to health care is restricted. Ectoparasite infestations and skin and respiratory tract infections have all been described as common in these detention centres where people are kept for as long as it takes to pay the traffickers' fees.^3^ Those able to pay attempt the Mediterranean crossing to Italy, a journey associated with the highest mortality of all routes to Europe in 2016, with one out of 47 people dying.^5^

**Research in context**

**Evidence before this study**

We searched PubMed using the search terms “whole genome sequencing”, “tuberculosis”, and “outbreak” for articles published in English up to Sept 1, 2017. Previous studies support whole-genome sequencing (WGS) as a method to direct *Mycobacterium tuberculosis* outbreak investigations with unprecedented resolution. However, it remains a new tool to which many high-income countries are only now considering a transition. Although its better resolution than more established typing methods has been repeatedly shown, the effect of WGS on international epidemiological investigations is only now being realised. We are not aware of it being used before in an as wide-ranging and complex investigation as described in this study.

**Added value of this study**

This analysis has provided further evidence of how WGS can both enrich and build on existing mycobacterial interspersed repetitive unit-variable number tandem repeat (MIRU-VNTR) data. First, among isolates sharing an MIRU-VNTR profile, it was able to distinguish those related through recent transmission from those with a distant common ancestor. Second, it has provided a clear example of how WGS data can be hypothesis generating with regards to transmission, especially its timing. Third, it provides an important example of how samples with matching MIRU-VNTR profiles, although not necessarily meeting the case definition, can be selected for sequencing to provide a genetic context for epidemiological analysis. The added value of the analysis is identification of what could be the first phylogenetic marker that confers drug resistance outside of that conferring intrinsic pyrazinamide resistance to *Mycobacterium bovis*. Such markers are of diagnostic value, while also providing the basis for rapid PCR assays that screen for further clustered isolates, a goal that the Network of the European Union, Latin America and the Caribbean Countries on Joint Innovation and Research Activities has lent its support to. Finally, our experience has shown that with ever-improving access to WGS platforms, these data can all be shared and analysed with ease across national boundaries. This approach shows the way forward to an improved European or wider international, cross-border alerting system for potential outbreaks. Standardisation of sequence data and metadata will no doubt facilitate data sharing across boundaries, helping public health practitioners, clinicians, and microbiologists who might wish to adjust diagnostic algorithms and techniques according to the bespoke requirements best fitting the epidemiological background of the local patient population.

**Implications of all the available evidence**

The benefits of WGS are now widely accessible. We have shown proof of principle that WGS can be incorporated contemporaneously into an international outbreak response. Although our efforts were somewhat ad hoc, facilitated by collaboration between university-based academics, state-level institutions, and the European Centre for Disease Prevention and Control, we are now at the point where WGS is ready to be incorporated into national and European standard programmes for cross-border identification, management, and prevention of tuberculosis outbreak scenarios.

Estimated tuberculosis incidence in the Horn of Africa ranges from 65 cases per 100 000 population per year in Eritrea to 192 cases per 100 000 per year in Ethiopia, 274 cases per 100 000 in Somalia, and 378 cases per 100 000 per year in Djibouti; Somalia has the highest estimated multidrug-resistant tuberculosis incidence, with 29 cases per 100 000 population per year.^1^ These numbers are only approximate^6^ and migration is expected to result in a higher incidence still among the subpopulation making the journey to Europe.^7^, ^8^ Of the many challenges for European authorities, early case finding and provision of adequate health care to this population are key to prevention of outbreaks.^9^, ^10^ In this study, we describe how a European response to a cluster of multidrug-resistant tuberculosis in patients from the Horn of Africa was jointly guided by rapid whole-genome sequencing (WGS) of *Mycobacterium tuberculosis* isolates, epidemiological investigations, and data sharing. The aim of the outbreak investigation was to elucidate the origin of the cluster, identify possible locations of transmission, and interrupt further transmission where possible.

## Methods

### Study design and participants

On April 29 and May 30, 2016, the Swiss and German National Mycobacterial Reference Laboratories independently triggered an outbreak investigation after four patients were diagnosed with multidrug-resistant tuberculosis. Isolates had matching 24-locus mycob-acterial interspersed repetitive unit-variable number tandem repeat (MIRU-VNTR) profiles and shared a rare *pncA* mutation. These patients had recently arrived from the Horn of Africa or Sudan. Retrospective and prospective data collection was initiated according to a case definition (inclusion criteria) based on mycobacteria growth indicator tube-based phenotypic resistance to isoniazid (≥3 mg/L), rifampicin (≥20 mg/L), ethambutol (2·5 mg/L to <5 mg/L), pyrazinamide (≥100 mg/L), and capreomycin (≥5 mg/L), with susceptibility to amikacin and fluoroquinolones (Loewenstein-Jensen media was used in France); and at least the shared MIRU-VNTR profile (multiple-locus variable number tandem repeat analysis MtbC 15–9: 5942–15)^11^ or associated mutations at *katG* S315T, *rpoB* S450L, *embB* M306I, *pncA* W68C, and *tlyA* N236K.

Investigations were done under local public health law in each country as part of an outbreak response. As such, no further ethical approval was necessary for most of the countries involved. For patients diagnosed in Switzerland, we obtained ethical approval and individual informed consent (Business Administration System for Ethics Committee ethics approval number 2016-02092).

### Procedures

The European Commission issued an Early Warning and Response System message to European Union member states after notification by the Swiss public health authorities of clustered cases in several European countries on Nov 17, 2016. Further suspected cases in Europe were reported to the European Centre for Disease Prevention and Control (ECDC) between Nov 17, 2016, and April 19, 2017. All isolates had WGS in their country of diagnosis or at the Research Centre Borstel, Borstel, Germany. All countries used Illumina (San Diego, CA, USA) platforms, except for Sweden, which used an Ion Torrent platform (Thermo Fisher Scientific, Waltham, MA, USA). We mapped short reads to the *M tuberculosis* reference (National Center for Biotechnology Information GenBank NC_000962.3) using Burrows-Wheeler Aligner version 0.7.12-r1039 and refined them using the Genome Analysis Toolkit and SAMtools. We used custom Perl scripts for variant-calling, with thresholds of a four-times coverage in both forward and reverse orientation, four-times coverage with a Phred score of at least 20, and 75% allele frequency. Variants in repetitive regions or genes were masked. We used only single-nucleotide polymorphisms (SNPs) for concatenated sequence alignments. Molecular clusters were defined for isolates with a minimum genome-wide distance of five SNPs to at least one other isolate. We prepared sequencing runs for a mean 20–50-times coverage, depending on the laboratory. We did statistical analyses in Stata (version 13.1). We deposited all sequences in the European Nucleotide Archive (accession PRJEB22358).

We investigated the molecular epidemiology following Strengthening the Reporting of Molecular Epidemiology for Infectious Diseases guidelines.^12^ To contextualise the outbreak against its genomic background, we sequenced additional MIRU-VNTR-matching isolates obtained from European countries' collections of isolates previously gathered from the Horn of Africa. Each month during the outbreak investigation, we built maximum likelihood (PhyML 3.1) and parsimony (Bionumerics 6.7) phylogenies to inform epidemiological investigations (appendix p 2).^13^

We did patient interviews in each country to establish migration routes and symptom onset dates. We reassured patients that interviews were confidential and independent of immigration authorities. We solicited certified translators if necessary. Structured questionnaires were used (appendix pp 3–27) and free narratives were recorded within them. Although each country's procedures differed, we gathered data for places of residence for 2 years preceding migration, route and means of migration, types of accommodation or detention centres, people they encountered en route, symptom onset, health-care seeking behaviour, and disease risk factors. We sought details of medical and treatment history from attending physicians in some countries. Whenever possible, we compared results with data from surveillance and screening systems. We reported results to the ECDC. When data were imprecise, we chose the first day of each month as the entry date into a new country where the month was given. We calculated each subsequent country visited in the same month as having been entered at 5-day intervals.

### Role of the funding source

With the exception of the Swiss Federal Office of Public Health (PH is an employee of the Swiss Federal Office of Public Health), the funders of the study had no role in study design, data collection, data analysis, data interpretation, or writing of the report. The corresponding author had full access to all the data in the study and had final responsibility for the decision to submit for publication.

## Results

29 patients fitting the case definition were identified across Europe between Feb 12, 2016, and April 19, 2017 (table). 14 (48%) were diagnosed in Germany, eight (28%) were diagnosed in Switzerland, two (7%) were each diagnosed in France and Austria, and one (3%) was each diagnosed in Sweden, Finland, and the UK. Before travel, the patients resided in Somalia (21 [72%] patients), Eritrea (three [10%]), Sudan (two [7%]), Ethiopia (two [7%]), and Djibouti (one [3%]); three (10%) patients were women and all were younger than 26 years of age (median 18 years [IQR 16·5–19·0]) at the time of diagnosis. One (3%) patient was lost to follow-up, with all others remaining on antituberculosis treatment under the care of the respective countries of their diagnosis. A further 29 patients whose isolates had related MIRU-VNTR types were identified after European collaborators searched their archives for matches; these cases all predated the outbreak (appendix pp 28, 29). Of these cases, 23 (79%) were isolated from patients resident in Europe at the time of diagnosis and six (21%) were isolated from patients resident in Djibouti. All patients originated from the Horn of Africa.

**Table:** Main characteristics of the 29 patients and susceptibility testing results of the outbreak multidrug-resistant tuberculosis isolates

	**Sex**	Age*	**Country of diagnosis**	**Country where journey started**	**Month of diagnosis**†	**Site of disease**	**Microscopy result**‡	**FASTQ accession number**	**Phenotypic drug susceptibility testing results (critical concentration testing)**																
									INH	RIF	EMB	PZA§	SM	MOX	OFL	AMI	KAN	CAP	ETH	PTH	CS	PAS	BDQ	DLM	LIN
AT1	Male	Child	Austria	Somalia	February, 2016	Pulmonary	Negative	ERS1927711	R	R	S	R	S	S	S	S	S	R	S	S	S	S	S	S	S
AT2	Male	Adult	Austria	Somalia	September, 2016	Pulmonary	Negative	ERS1927712	R	R	S	R	S	S	S	S	S	R	S	S	S	S	S	S	S
DE1	Male	Child	Germany	Somalia	March, 2016	Pulmonary	Negative	ERS1927720	R	R	S	R	S	S	NE	S	NE	R	NE	NE	NE	NE	NE	NE	NE
DE2	Male	Child	Germany	Somalia	March, 2016	Pulmonary	Negative	ERS1927721	R	R	S	R	NE	NE	S	S	NE	R	NE	NE	NE	NE	NE	NE	NE
DE3	Female	Adult	Germany	Somalia	March, 2016	Pulmonary	Negative	ERS1927722	R	R	S	R	S	S	NE	S	NE	R	NE	NE	NE	NE	NE	NE	NE
DE4	Male	Child	Germany	Somalia	March, 2016	Pulmonary	Positive	ERS1927723	R	R	S	R	S	NE	S	S	NE	R	NE	NE	NE	NE	NE	NE	NE
DE5	Male	Adult	Germany	Somalia	July, 2016	Pulmonary	Negative	ERS1927724	R	R	S	R	NE	NE	S	S	NE	R	NE	NE	NE	NE	NE	NE	NE
DE6	Male	Adult	Germany	Somalia	August, 2016	Pulmonary	Negative	ERS1927725	R	R	S	R	S	NE	S	S	S	R	NE	NE	NE	NE	NE	NE	NE
DE7	Male	Adult	Germany	Somalia	November, 2016	Pulmonary	Positive	ERS1927726	R	R	R	R	S	S	S	S	S	R	NE	NE	NE	NE	NE	NE	NE
DE8	Male	Child	Germany	Somalia	September, 2016	Pulmonary	Positive	ERS1927727	R	R	NE	R	NE	NE	NE	S	NE	R	NE	NE	NE	NE	NE	NE	NE
DE9	Male	Child	Germany	Somalia	May, 2016	Pulmonary	Negative	ERS1927728	R	R	R	R	S	S	S	S	S	R	NE	NE	NE	NE	NE	NE	NE
DE10	Male	Adult	Germany	Somalia	March, 2016	Pulmonary	Negative	ERS1927715	R	R	NE	R	S	S	S	S	S	R	NE	NE	NE	NE	NE	NE	NE
DE11	Male	Child	Germany	Somalia	May, 2016	Pulmonary	Negative	ERS1927716	R	R	R	R	NE	NE	NE	S		R	NE	NE	NE	NE	NE	NE	NE
DE12	Male	Adult	Germany	Eritrea	November, 2016	Pulmonary	Negative	ERS1927717	R	R	R	R	S	S	S	S	S	R	NE	NE	NE	NE	NE	NE	NE
DE13	Female	Child	Germany	Somalia	December, 2016	Pulmonary	Negative	ERS1927718	R	R	NE	R	NE	NE	NE	S	NE	R	NE	NE	NE	NE	NE	NE	NE
DE14	Male	Adult	Germany	Somalia	November, 2016	Pulmonary	Positive	ERS1927719	R	R	R	R	S	S	S	S	S	R	NE	NE	NE	NE	NE	NE	NE
FI1	Male	Adult	Finland	Eritrea	October, 2016	Pleural	Negative	ERS1927735	R	R	NE	NE	S	S	S	S	S	R	S	NE	NE	NE	NE	NE	NE
FR1	Male	Adult	France	Somalia	April, 2016	Pulmonary	Negative	ERS1927739	R	R	NE	NE	S	S	S	S	S	R	S	NE	NE	NE	S	NE	NE
FR2	Male	Adult	France	Sudan	October, 2016	Pulmonary	Positive	ERS1927740	R	R	NE	NE	S	S	S	S	S	R	S	NE	NE	NE	S	NE	NE
SE1	Female	Child	Sweden	Somalia	August, 2016	Pulmonary	Negative	ERS1927768	R	R	NE	NE	NE	S	S	S	S	R	S	NE	NE	NE	NE	NE	NE
SW1	Male	Adult	Switzerland	Somalia	December, 2015	Intrathoracic lymph nodes	Negative	ERS1927751	R	R	S	R	S	S	NE	S	S	R	S	NE	S	S	S	S	S
SW2	Male	Adult	Switzerland	Somalia	April, 2016	Pulmonary	Negative	ERS1927752	R	R	S	R	S	S	NE	S	S	R	S	NE	S	S	S	S	S
SW3	Male	Child	Switzerland	Somalia	May, 2016	Pulmonary	Positive	ERS1927753	R	R	S	R	S	S	NE	S	S	R	S	NE	S	S	S	S	S
SW4	Male	Adult	Switzerland	Eritrea	May, 2016	Pulmonary	Positive	ERS1927754	R	R	S	R	S	S	NE	S	S	R	S	NE	S	S	S	S	S
SW5	Male	Child	Switzerland	Ethiopia	June, 2016	Pulmonary	Negative	ERS1927755	R	R	S	R	S	S	NE	S	S	R	S	NE	S	S	S	S	S
SW6	Male	Adult	Switzerland	Somalia	April, 2016	Pulmonary	Positive	ERS1927756	R	R	S	R	S	S	NE	S	S	R	S	NE	S	S	S	S	S
SW7	Male	Adult	Switzerland	Ethiopia	August, 2016	Pulmonary	Negative	ERS1927757	R	R	S	R	S	S	NE	S	S	R	S	NE	S	S	S	S	S
SW8	Male	Adult	Switzerland	Djibouti	September, 2016	Pleural	Negative	ERS1927758	R	R	S	R	S	S	NE	S	S	R	S	NE	S	S	S	S	S
UK1	Male	Adult	UK	Sudan	April, 2017	Spinal	Unknown	ERS1927759	R	R	R	S	S	S	S	S	S	R	NE	S	NE	S	NE	NE	S

INH=isoniazid. RIF=rifampicin. EMB=ethambutol. PZA=pyrazinamide. SM=streptomycin. MOX=moxifloxacin. OFL=ofloxacin. AMI=amikacin. KAN=kanamycin A. CAP=capreomycin. ETH=ethionamide. PTH=prothionamide. CS=cycloserine. PAS=para-aminosalicylic acid. BDQ=bedaquiline. DLM=delamanid. LIN=linezolid. R=resistant. S=susceptible. NE=not established.

* A child is defined as younger than 18 years of age.

† According to the state where the patient was first diagnosed.

‡ Qualitative result of fluorescence direct material microscopy (auramine-rhodamine staining).

§ All outbreak isolates had the *pncA* W68C mutation.

**Figure 1:**
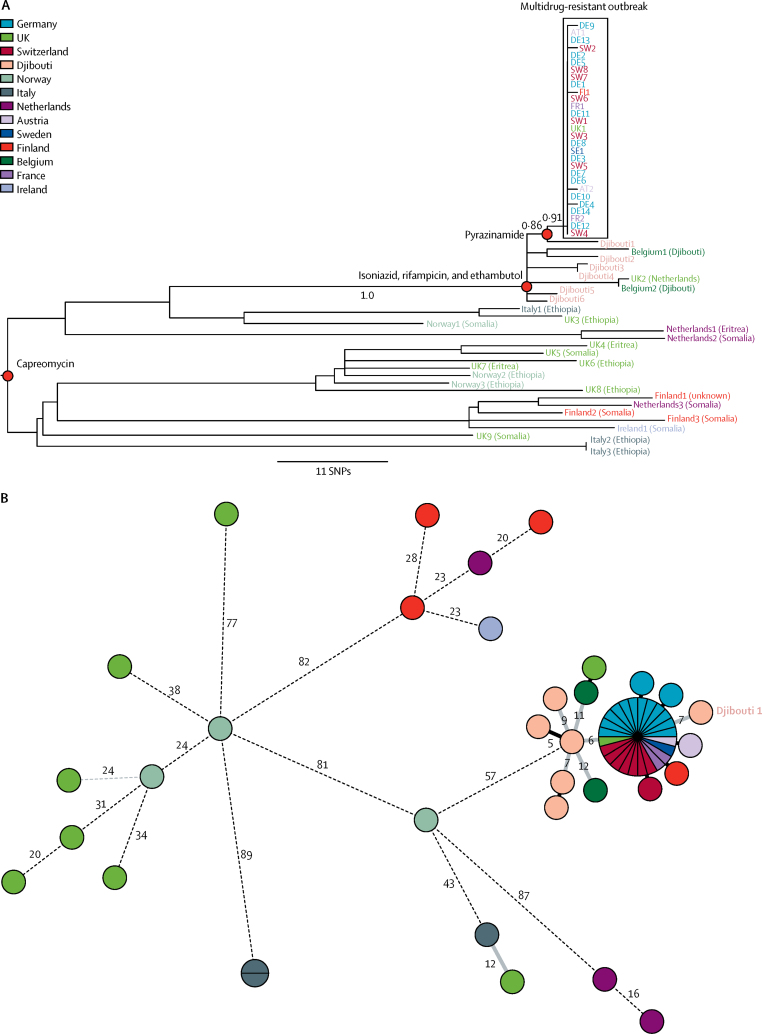
Genetic relationship or distances between 58 *Mycobacterium tuberculosis* isolates associated with a multidrug-resistant tuberculosis outbreak among migrants in 2016 and 2017 in Europe Maximum likelihood phylogeny (A) and minimum spanning tree (B). Numbers in B show genetic distances in SNP differences and solid black branches without annotation reflect 1 SNP difference. SNP=single-nucleotide polymorphism.

WGS analysis identified 549 nucleotide variants and placed the 58 samples into sublineage 4.6.2 (Cameroon genotype), part of the European-American lineage.^14^ Any two of the original 29 outbreak isolates were separated by a maximum of two SNPs, whereas the 29 other samples were separated by more than five SNPs, and in 18 cases, by more than 100 SNPs (figure 1). These additional isolates enabled a phylogenetic analysis of the stepwise evolution of drug resistance mutation characteristics for this cluster. Whereas resistance to isoniazid, rifampicin, and ethambutol was ancestral to both the outbreak samples and all of the outlier samples from Djibouti, pyrazinamide resistance, which completed the original outbreak case definition, was acquired later. In addition to all of the original outbreak samples, pyrazinamide resistance was limited to just a single isolate from the Djibouti outlier samples, which was seven SNPs away from the main cluster (figure 1). All 58 isolates shared the *tlyA* N236K mutation and underlying resistance to capreomycin,^15^, ^16^ but none were resistant to the aminoglycosides. 45 (96%) of 47 isolates tested for capreomycin resistance were indeed phenotypically resistant, including isolates that were susceptible to all antituberculosis drugs, suggesting that this mutation could be both a phylogenetic marker and a drug resistance mutation.

Considering molecular clock estimates for *M tuberculosis* of around 0·3–0·5 SNPs per genome per year,^17^, ^18^, ^19^, ^20^ the degree of genomic homogeneity among the 29 outbreak isolates (figure 1) suggests recent transmission. Interviews therefore focused especially on establishing locations of likely transmission. One (3%) patient was lost to follow-up, and travel details for six (21%) were unavailable. Of the 22 (76%) patients for whom data were available, all reported making the journey across the Mediterranean from Libya to Italy, and none described a plausible alternative route. Because patients originated from different locations within different countries and because of the range of countries in which these journeys ended, patients are most likely to have met en route through Sudan, Libya, or Italy.

**Figure 2:**
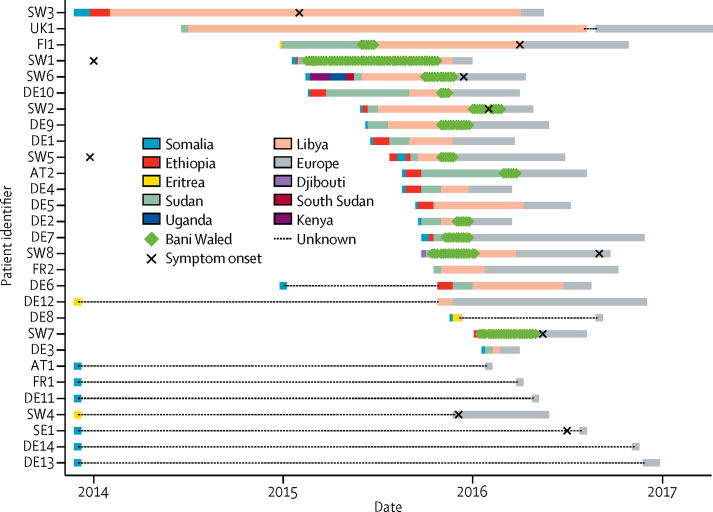
Timeline of patient journeys until diagnosis The 1st of each month is shown as the country entry date. Subsequent countries visited in the same month are presented at 5-day intervals.

17 (77%) patients reported transiting through Sudan, with a 15 day median length of stay (IQR 5–30), but few further details about patients' stays in Sudan were available. All 22 patients for whom data were available recounted transiting through Libya (median 100 days [60–180]), where at least 12 were held by human traffickers for extended periods of time. Libya's main bottleneck appears to have been the town of Bani Waleed, where the median length of stay was longer (53 days [30–73]) than the entire time spent in Sudan. 12 (55%) of 22 patients either named Bani Waleed, 180 km southeast of Tripoli and 135 km southwest of Misrata, as the location of their detention, or recounted a detention centre matching its description.^3^ Conditions were described as overcrowded and unsanitary. Several patients reported that they were detained with about 300 other people in a closed hall or hangar, with a roof and no windows. Multiple reports emerged of physical abuse by the captors and widespread disease and coughing. Of the 12 patients likely to have stayed in Bani Waleed between February, 2015, and May, 2016, each overlapped with at least one other, and five overlapped at once in November, 2015 (figure 2). Six (50%) of these patients were eventually diagnosed in Switzerland, four (33%) were diagnosed in Germany, and one (8%) was each diagnosed in Austria and Finland. All countries responded to the outbreak by providing patients with multidrug-resistant tuberculosis treatment, including hospital admission where necessary, and did contact investigations to prevent further transmission.

The most detailed epidemiological information was available for seven of eight patients diagnosed in Switzerland (SW1–3 and SW5–8). Among these seven patients, five mentioned passing through Bani Waleed and one described a location consistent with it. We considered whether or not any of these cases could have been the outbreak source case. SW1, who was the first known outbreak case to arrive in Bani Waleed, lived approximately 60 km south of Hargeisa in northern Somalia. Although he reported having haemoptysis in 2014 before migration, he reported no further symptoms between 2014 and arrival in Switzerland. SW5, who lived approximately 80 km from Hargeisa, over the Ethiopian border, also reported haemoptysis in 2014 before migration. However, he was eventually diagnosed with mediastinal lymph node disease in Switzerland, where no evidence of parenchymal lung disease was identified to account for the history of haemoptysis. Both patients passed through Hargeisa at different times, one spending 2 months and the other spending 10 days there.

An alternative candidate source case was SW8, who grew up in a refugee camp in Djibouti more than 200 km northwest of Hargeisa and reported a family tuberculosis contact before migration. However, he was eventually diagnosed with pleural tuberculosis and therefore not considered infectious. SW2 came from Mogadishu where he reported a family tuberculosis contact. Although he passed through Hargeisa, he only stayed for one night. Although his symptoms are thought to have started in Bani Waleed, he arrived there after other outbreak patients had already arrived in Europe (figure 2), so he could not have been the source. SW6 originated from Mogadishu and travelled with a cousin who died with haemoptysis shortly after they both left Bani Waleed. Assuming that they travelled together all the way, they would have arrived too late to have infected FI1, the first of the known outbreak patients to leave Bani Waleed (figure 2). FI1 was never infectious himself. More detailed data for other patients passing through Bani Waleed were not available.

**Figure 3:**
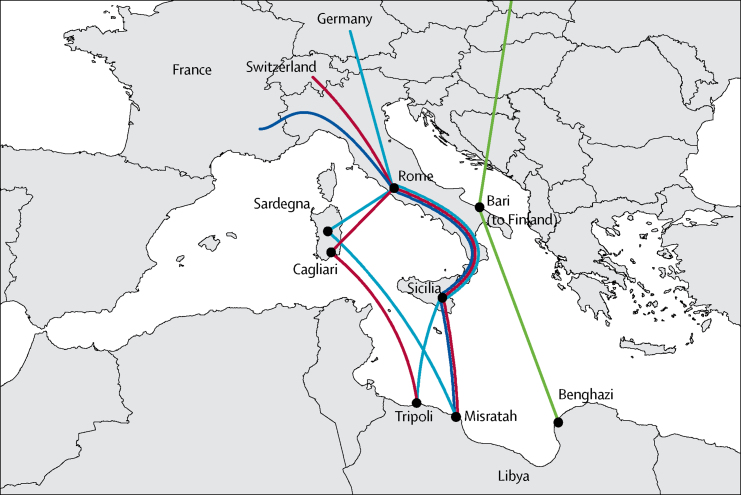
Reported migration routes through Italy of 29 outbreak patients with a documented migration route, August, 2014, to February, 2017

Italy's bottleneck was wider, with various ports of disembarkation described, including Lampedusa, mainland Sicily, Sardinia, and Apulia (figure 3). Further details of when patients passed through other Italian cities and the location and conditions in which they stayed were not available, making inferences about the likelihood of transmission between individuals in Europe difficult. So far, no cases of transmission to people already living in Europe have been reported, and a search of the ECDC MIRU-VNTR-based database of isolates covering the period of 2003–15 identified only those isolates that we had already included as outliers.

## Discussion

This investigation involved a coordinated effort across seven European countries over 6 months. A process of local sequencing of isolates and centralised analysis of WGS data in Borstel was rapidly coordinated to confirm patients' links to the cluster. Epidemiological data were simultaneously collected at a country level and shared with national tuberculosis surveillance programmes, the ECDC, and the international team of researchers aiding the analysis. All patients remain on treatment under the supervision of local health-care teams, with the exception of one who was lost to follow-up. No reports have emerged of transmission of this outbreak strain to people other than those making the perilous journey from the Horn of Africa or Sudan to Europe.

Somalis made up just 8·1% of migrants or refugees crossing the Mediterranean northwards in 2015,^9^ but 22 (76%) of 29 patients in the outbreak, suggesting social clustering along cultural or linguistic lines as patients travelled. From the available epidemiological data, the most likely hotspot of transmission is Bani Waleed, where 12 patients are thought to have passed through; additionally, transmission is likely to have happened elsewhere along the route to account for the other genomically tightly clustered cases. Given the low genomic variation between the outbreak isolates, the most parsimonious interpretation is that a single introduction of the strain to Bani Waleed occurred or several patients were infected en route before arrival or shortly after leaving. The alternative hypothesis that geographically dispersed patients whose *M tuberculosis* isolates share a very recent ancestor were infected before starting their travels within the same time window is most unlikely.

Identification of the likely source case is difficult. Although other patients might well have been involved, among the patients for whom data were available, SW1 might be the most likely source if one assumes that he was symptomatic en route, despite what he reported. This hypothesis would be consistent with the phylogeographical signal in the sequence data that shows that all of the serendipitously obtained outlier isolates—ie, those from Djibouti—share the closest common ancestor with the outbreak isolates. The other outlier isolates originate from a wider area than Djibouti and are likely to represent a larger regional clonal expansion of a strain that we have subsampled here.

A major limitation of the epidemiological data is that patient interviews were, by their nature, retrospective. The refugees or migrants might easily have lost sense of both time and location during their journeys and their accounts might have lacked precision. They often travelled at night, in trucks, under direction of human traffickers, held in detention camps without access to communications. Moreover, some patients were uncomfortable giving an accurate account of their journey even if they did recall it. This reluctance might have been due to the trauma—accounts emerged of executions, rape, forced labour, and refugees being sold into slavery by the human traffickers—or to fear of the data being shared with immigration authorities and it affecting their asylum claims. The main limitations of the genomic data were the few available contextualising isolates and the fact that they were obtained from a convenience sample.

The five-SNP threshold that separated our outbreak isolates from the outliers was calibrated in a low-burden setting where it was considered predictive of recent transmission. However, in a high-intensity transmission setting, a lower threshold than this one might be warranted.^18^, ^21^ The fact that a maximum of two SNPs separate any two outbreak isolates does, however, suggest a recent common ancestor, if not necessarily direct transmission between patients. The existence of additional perhaps intermediary cases unknown to us somewhere between the Horn of Africa and Europe is very likely.

The presence of the *tlyA* N236K mutation in all 58 samples, both multidrug-resistant and susceptible, suggests that this mutation is a phylogenetic marker for this sub-branch of the Cameroon sublineage. Further sampling will be required to assess how deep this mutation lies on the tree. Although it was not featured by Coll and colleagues^14^ as part of their SNP barcode for typing of *M tuberculosis*, a distance of more than 100 SNPs between the most remotely related of the 58 isolates suggests a common ancestor likely to predate the antibiotic era. An allelic exchange experiment would be one way to definitively explore whether or not this SNP is the first phylogenetic marker in *M tuberculosis* sensu stricto underlying resistance to an antituberculosis drug, but now substantial supporting circumstantial evidence exists. Not only were 45 (96%) of 47 tested isolates resistant to capreomycin, regardless of their susceptibility to other drugs, but also isolates were simultaneously susceptible to amikacin and kanamycin. Variation in *tlyA* is specific to capreomycin resistance and unlike variation in *rrs* does not also confer resistance to the aminoglycosides. Since this mutation was the only non-synonymous mutation within *tlyA* and *rrs*, the case appears strong.^16^, ^22^, ^23^ In the absence of selection through a historically indiscriminate use of capreomycin in the Horn of Africa, which we believe to be unlikely, this mutation is likely to have arisen through genetic drift, only coincidentally leading to capreomycin resistance. Its fixation implied absence of fitness cost.^24^, ^25^ All multidrug-resistant tuberculosis isolates from this sublineage should therefore by definition be considered pre-extensively drug resistant.

More refugees and migrants are expected to attempt the Mediterranean crossing from Libya in summers to come, increasing the chances of seeing additional cases linked to this cluster in Europe. Until mid-July, 2017, at the time of writing, four additional isolates derived from patients diagnosed in Germany and one from a patient diagnosed in Italy, with the characteristic antibiotic susceptibility profile, were confirmed as part of the outbreak by WGS analysis. Each had a maximum number of two SNPs difference to any other outbreak isolate. Because of the unstable political situation in Libya, direct public health intervention was not possible in what is most likely to have been the main hotspot of transmission in this outbreak. The chances of achievement of this intervention in northern Somalia or Djibouti might be marginally greater than in Libya, but clearly still very challenging. However, this investigation has served to heighten awareness of the small but consequential risk of multidrug-resistant tuberculosis in this population, and linkage of patients to what might be an ongoing reservoir of source cases in Bani Waleed or elsewhere will nevertheless be useful. An understanding that ongoing transmission might be occurring in such centres will inform plans for more targeted screening and follow-up of patients who report passing through them than have been done so far. The investigation has also provided the data from which a rapid PCR-based screening test can be designed for laboratories serving the refugee and migrant reception centres for whom WGS platforms might still be out of reach. The mutations in *pncA* and *tlyA* could easily be incorporated into such a screening test that could be used alongside the usual commercial tests, such as the Xpert MTB/RIF test (Cepheid, Sunnyvale, CA, USA), as identification and treatment of multidrug-resistant tuberculosis is of primary importance, whatever the strain. Indeed, a parallel E-detect TB initiative has already stationed the Xpert test in Sicily for migrant screening, with a plan to whole-genome sequence all multidrug-resistant tuberculosis cases identified and improve on some of the previously identified shortcomings in screening.^26^

A molecular epidemiological approach synthesising WGS and epidemiological data has helped delineate a multidrug-resistant tuberculosis outbreak against the broad setting of a clonal expansion of a strain of *M tuberculosis* in the Horn of Africa and informed the hypothesis that this strain might have emerged in northern Somalia or Djibouti, with ongoing transmission in a detention camp in Libya. The WGS data have incidentally also helped identify what we hypothesise is the first phylogenetic marker in *M tuberculosis* sensu stricto that also causes drug resistance. By contrast, MIRU-VNTR data were not able to distinguish between outbreak and background isolates. This outbreak has shown how high-resolution WGS data can be produced rapidly, across borders, through effective collaboration between microbiological laboratories and national and supranational institutions, to good effect. To optimise the gain from such technology, it needs to be integrated as a routine component of outbreak response efforts. Consequently, the ECDC is now piloting a European Union-wide sequencing study (EUSeqMyTB),^27^ with an initial focus on multidrug-resistant tuberculosis. Provision of a supportive environment allowing patients to adhere to treatment without fear of deportation is equally important, however.
