# Corrections

**DOI:** 10.1016/S1473-3099(18)30024-0

**Published:** 2018-03

*Walker TM, Merker M, Knoblauch AM, et al. A cluster of multidrug-resistant Mycobacterium tuberculosis among patients arriving in Europe from the Horn of Africa: a molecular epidemiological study*. Lancet Infect Dis *2018 published online Jan 8. http://dx.doi.org/10.1016/S1473-3099(18)30004-5*—In this Article, the appendix file has been replaced with the correct version. This correction has been made to the online version as of Jan 10, 2018.

