# Supplementary material for: A cluster of multidrug-resistant Mycobacterium tuberculosis among patients arriving in Europe from the Horn of Africa: a molecular epidemiological study
Source: Lancet Infect Dis. 2018 Apr;18(4):431–40. doi: 10.1016/S1473-3099(18)30004-5 (PMC5864516; doi:10.1016/S1473-3099(18)30004-5)
Supplement: Supplementary appendix [file mmc1.pdf]

THE LANCET Infectious Diseases

Supplementary webappendix

This webappendix formed part of the original submission and has been peer reviewed. We post it as supplied by the authors.

This online publication has been corrected. The corrected version first appeared at thelancet.com/infection on Jan 10, 2018.

Supplement to: Walker TM, Merker M, Knoblauch AM, et al, and the MDR-TB Cluster Consortium. A cluster of multidrug-resistant *Mycobacterium tuberculosis* among patients arriving in Europe from the Horn of Africa: a molecular epidemiological study. *Lancet Infect Dis* 2018; published online Jan 8. http://dx.doi.org/10.1016/S1473-3099(18)30004-5.

Supplementary appendix

Supplement to: Walker TM, Merker M, Knoblauch A, et al. A cluster of multi-drug resistant *Mycobacterium tuberculosis* among patients arriving in Europe from the Horn of Africa: A molecular epidemiological study.

Table of contents

Supplementary Methods 2

Calculation of the maximum likelihood phylogeny 2

Calculation of the minimum spanning tree (MST) 2

Patient questionnaire (English version) 3

Patient questionnaire (German/French version) 15

Supplementary Table 1 28

Supplementary Methods

Calculation of the maximum likelihood phylogeny.

The tree was calculated with PhyML 3.1 utilizing 549 variable genome positions among all isolates and a Generalised Time Reversible (GTR) substitution model. Branches differentiating the identified MDR-TB outbreak from other lineage 4.6.2 MTB isolates are labelled with confidence values based on 100 bootstrap replicates. Step-wise resistance acquisition to individual first- and second-line anti-TB drugs mediated by distinct mutations, and leading to the characteristic resistance profile of the investigated outbreak (black box) indicated on ancestral nodes (red dots).

Calculation of the minimum spanning tree (MST).

The tree was calculated with BioNumerics 7.5 utilizing the identical sequence alignment as used for Figure 1 (549 variable positions (SNPs), respectively the derived distance matrix). The algorithm considers the minimum evolution criteria and reduces the total distance between all samples to a minimum. Number of SNPs differentiating two samples annotated on branches; solid black branches without annotation reflect 1 SNP difference. Compared to a maximum likelihood approach (see Figure 1) no hypothetical (ancestor) states are introduced in a MST, which aligns the Djibouti1 strain directly to the MDR-outbreak, which comprise 24 identical isolates and 5 one-SNP variants. The link between Djibouti1 and the MDR-outbreak can be interpreted as a result of sampling limitations among outlier strains rather than reflecting a direct phylogenetic relationship, i.e. patient-to-patient transmission.

Patient questionnaire – English version

Published with kind the permission of the Swiss Federal Office of Public Health.

**Patient Questionnaire**		
**A. Information for the patient**		
In Switzerland, in 2016, eight cases of multidrug-resistant tuberculosis (MDR-TB) were diagnosed that belong to the same cluster. Further cases from the same cluster were diagnosed in other European countries and therefore a pan-European investigation was initiated. In Switzerland, on 29 November 2016, the Center for Epidemiological Outbreak Investigations (KEA), led by the Swiss Tropical and Public Health Institute (Swiss TPH), was commissioned by the Swiss Federal Office of Public Health (FOPH) to conduct an epidemiological investigation of the cases. The investigation is based on Article 20 of the Registration Ordinance (Meldeverordnung) and on the basis of the Swiss Law on Epidemics.<br>Since the primary objective of the study is to identify the site(s) of infection and the pathways of infection, places of origin of cases, migration routes and places of stay should be defined as precisely as possible geographically and in terms of time periods.<br>The information collected during this interview will be kept strictly confidential and will be accessible only to the study investigators. In particular, this information is not sent to the migration authorities. The questionnaire will be kept in anonymised form, giving you an identification number and your name will not be mentioned or noted down. You have the freedom to answer questions without giving reasons.		
**B. General information**		**Comments**
**1**	Patient ID:	
**2**	Do you agree that the conversation will be recorded?<br>☐1 Yes<br>☐2 No	
**3**	Languages of the interview:<br>☐1 German<br>☐2 French<br>☐3 Italian<br>☐4 English<br>☐5 Somali<br>☐6 Tigrinya<br>☐7 Other(s): ____________________________	
**4**	Translation:<br>☐1 No<br>☐2 Yes (specify): _________________________	
**5**	Persons present at the interview:<br>☐1 Patient<br>☐2 KEA employee<br>☐3 Employee of the Swiss Lung Association<br>☐4 Translator<br>☐5 Other: __________________________	
**6**	Sex of the patient:<br>☐1 Male<br>☐2 Female	
**7**	Canton of current residence:<br>☐1 St. Gallen<br>☐2 Ticino<br>☐3 Vaud	

	☐4 Zürich	
**8**	Were you before or after accommodated in other cantons or in other accommodations in Switzerland?<br>☐1 Yes<br>☐2 No→ *10*<br>☐99 Don't know, unknown→ *10*	*See case listing*
**9**	If yes, where and when?	
**C. Demographic information and migration route**		
**10.1**	Date of birth: *(DD/MM/YYYY)*<br>______________________________<br>☐99 Don't know / Refused	
**10.2**	Age in years:<br>______________________________<br>☐99 Don't know / Refused	
**11**	Can you tell us exactly where you were born?<br>Country: ____<br>Region: ____<br>Village/town: ____<br>☐99 Don't know / Refused	
**12**	Can you name the locations and time periods where you lived <u>during the last 2 years before leaving your country</u>?	**Country where the migration journey started.*

**Time period** ***(DD/MM/YYYY)***	**Country**	**Location: town/village**

The following maps can be used with questions 13.0-13.14 to reconstruct the migration route.

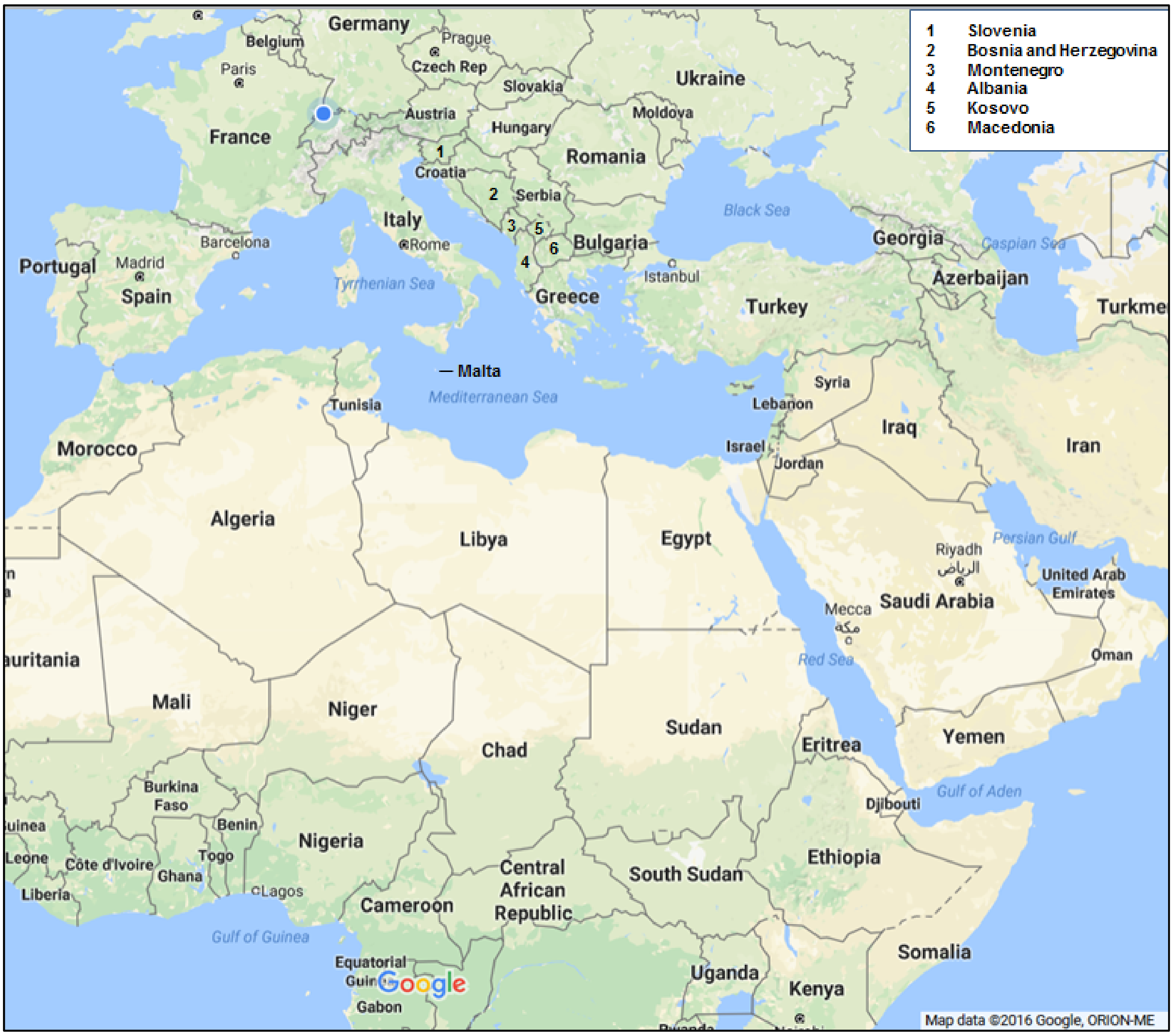
1 Slovenia
2 Bosnia and Herzegovina
3 Montenegro
4 Albania
5 Kosovo
6 Macedonia
Germany
Belgium
Prague
Czech Rep
Paris
France
Slovakia
Austria
Hungary
Ukraine
Moldova
Romania
Croatia
Serbia
Italy
Rome
Bulgaria
Black Sea
Georgia
Caspian Sea
Azerbaijan
Istanbul
Portugal
Madrid
Spain
Barcelona
Tyrrhenian Sea
Greece
Turkey
Turkmen
Malta
Mediterranean Sea
Tunisia
Syria
Lebanon
Iraq
Iran
Israel
Jordan
Morocco
Algeria
Libya
Egypt
Riyadh
Persian Gulf
United Arab Emirates
Mecca
Saudi Arabia
Red Sea
Oman
Mali
Niger
Chad
Sudan
Eritrea
Yemen
Burkina Faso
Benin
Togo
Nigeria
Djibouti
Gulf of Aden
Ethiopia
Côte d'Ivoire
Ghana
Liberia
Lagos
Cameroon
Central African Republic
South Sudan
Somalia
Gulf of Guinea
Equatorial
Gabon
Uganda
Kenya
Google
Map data ©2016 Google, ORION-ME

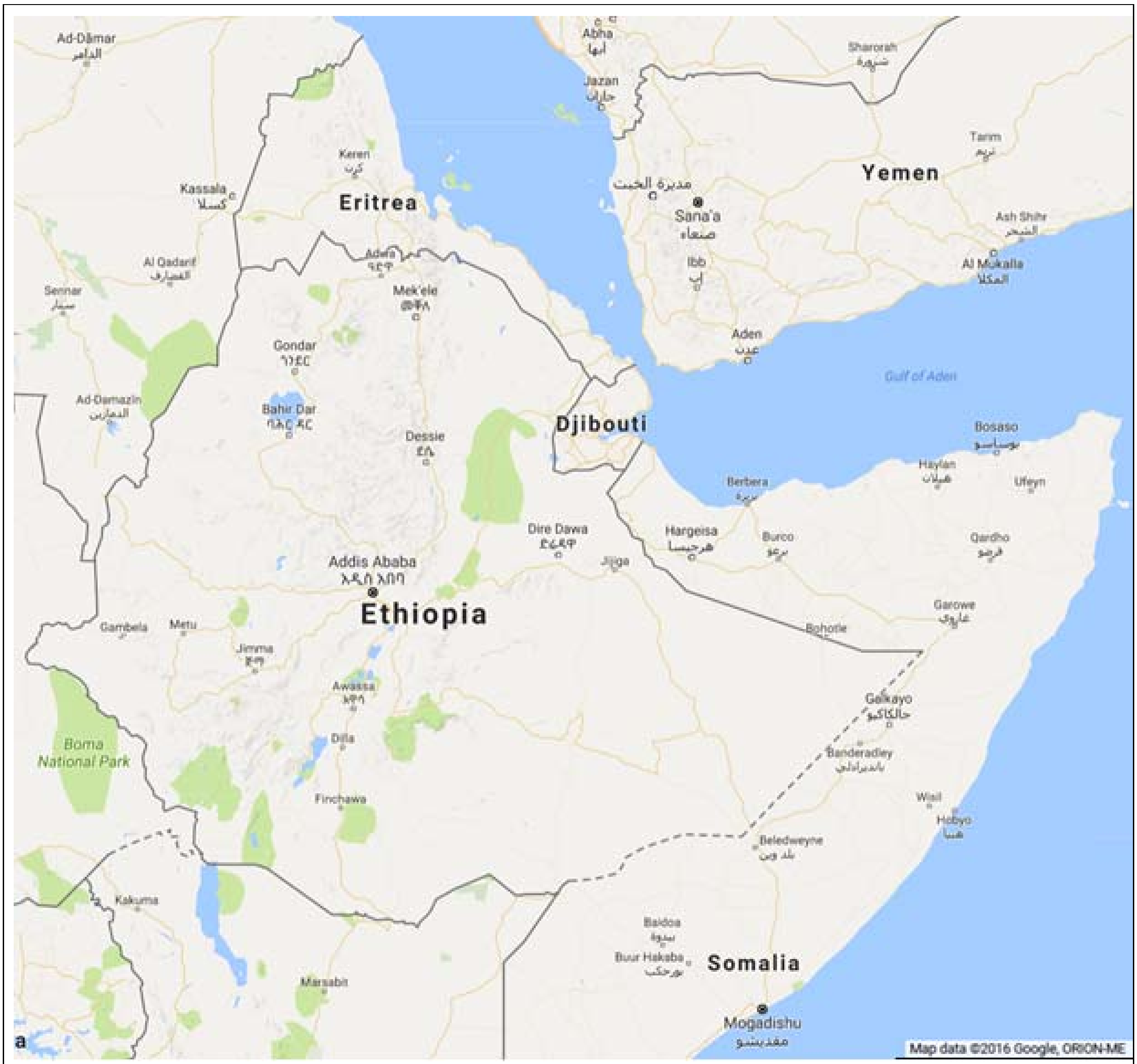
Eritrea
Ethiopia
Djibouti
Yemen
Somalia
Gulf of Aden
Boma National Park
Ad-Dāmar
Kassala
Al Qadarif
Sennar
Ad-Damazin
Keren
Adwa
Mek'ele
Gondar
Bahir Dar
Dessie
Addis Ababa
Dire Dawa
Jijiga
Gambela
Metu
Jimma
Awassa
Dilla
Finchawa
Kakuma
Marsabit
Abha
Jazan
Sharorah
Tarim
Sana'a
Ibb
Aden
Ash Shihr
Al Mukalla
Bosaso
Haylan
Ufeyn
Berbera
Hargeisa
Burco
Qardho
Garowe
Bohotle
Galkayo
Banderadley
Wisil
Hobyo
Beledweyne
Baidoa
Buur Hakaba
Mogadishu
Map data ©2016 Google, ORION-ME

13	We now want to reconstruct your migration route in chronological order. You can talk freely about your migration route.<br>• If possible, please tell us the travel routes from one place to another, including the stopovers and stays in those places and associated time periods.<br>• If possible, please also tell us what modes of transport used or the type of accommodation stayed in, the name of the accommodation and how many people you were staying, if you remember.<br>Question: When you started your migration journey to *"(the last mentioned place of residence in question 12)"*, when exactly did you leave this place (DD/MM/YYYY, as accurately as possible) and where did your journey go from there?<br><br>*Note: Continue to ask questions for each step of the journey. Any trip interrupted by a stopover/stay or change of modes of transport should be listed as a new, separate step.*<br><br>*[1]Institution [e.g. Asylum seeker reception center; transit center, home for asylum seekers (house or apartment); homeless shelter]*

			**Journey**		**Stopover**		
	**a. Time period** *(DD/MM/YY)*	**b. Journey or stopover?**	**c. Route** *(Destination A – Destination B)*	**d. Mode of transport**	**e. Place of stayover** *(Country and town)*	**f. Type of accommodation (acc.)**	**g. Name of accommodation, # of co-habitants**
**13.1**	☐$_{99}$ Don't know	☐$_1$ Journey → *13c&d*<br>☐$_2$ Stopover → *13e-g*<br>☐$_3$ Other: ___________<br>☐$_{99}$ Don't know	Destination A: ___________<br>Destination B: ___________<br>☐$_{99}$ Don't know	☐$_1$ Plane<br>☐$_2$ Car, bus<br>☐$_3$ Boat<br>☐$_4$ By foot<br>☐$_5$ Animal<br>☐$_6$ Other: ___________<br>☐$_{99}$ Don't know	Country: ___________<br>Ville: ___________<br>☐$_{99}$ Don't know	☐$_1$ Private acc.<br>☐$_2$ Refugee camp<br>☐$_3$ Institution[1]<br>☐$_4$ Prison<br>☐$_5$ Hospital<br>☐$_6$ None, homeless<br>☐$_7$ Hotel<br>☐$_8$ Other: ___________<br>☐$_{99}$ Don't know	☐$_{99}$ Don't know
**13.2**	☐$_{99}$ Don't know	☐$_1$ Journey → *13c&d*<br>☐$_2$ Stopover → *13e-g*<br>☐$_3$ Other: ___________<br>☐$_{99}$ Don't know	Destination A: ___________<br>Destination B: ___________<br>☐$_{99}$ Don't know	☐$_1$ Plane<br>☐$_2$ Car, bus<br>☐$_3$ Boat<br>☐$_4$ By foot<br>☐$_5$ Animal<br>☐$_6$ Other: ___________<br>☐$_{99}$ Don't know	Country: ___________<br>Ville: ___________<br>☐$_{99}$ Don't know	☐$_1$ Private acc.<br>☐$_2$ Refugee camp<br>☐$_3$ Institution[1]<br>☐$_4$ Prison<br>☐$_5$ Hospital<br>☐$_6$ None, homeless<br>☐$_7$ Hotel<br>☐$_8$ Other: ___________<br>☐$_{99}$ Don't know	☐$_{99}$ Don't know

	a. Time period *(DD/MM/YY)*	b. Journey or stopover?	Journey		Stopover		
			c. Route *(Destination A – Destination B)*	d. Mode of transport	e. Place of stayover *(Country and town)*	f. Type of accommodation (acc.)	g. Name of accommodation, # of co-habitants
**13.3**	☐99 Don't know	☐1 Journey → *13c&d*<br>☐2 Stopover → *13e-g*<br>☐3 Other: ____________<br>☐99 Don't know	Destination A: ____________<br>Destination B: ____________<br>☐99 Don't know	☐1 Plane<br>☐2 Car, bus<br>☐3 Boat<br>☐4 By foot<br>☐5 Animal<br>☐6 Other: ____________<br>☐99 Don't know	Country: ____________<br>Ville: ____________<br>☐99 Don't know	☐1 Private acc.<br>☐2 Refugee camp<br>☐3 Institution[1]<br>☐4 Prison<br>☐5 Hospital<br>☐6 None, homeless<br>☐7 Hotel<br>☐8 Other: ____________<br>☐99 Don't know	☐99 Don't know
**13.4**	☐99 Don't know	☐1 Journey → *13c&d*<br>☐2 Stopover → *13e-g*<br>☐3 Other: ____________<br>☐99 Don't know	Destination A: ____________<br>Destination B: ____________<br>☐99 Don't know	☐1 Plane<br>☐2 Car, bus<br>☐3 Boat<br>☐4 By foot<br>☐5 Animal<br>☐6 Other: ____________<br>☐99 Don't know	Country: ____________<br>Ville: ____________<br>☐99 Don't know	☐1 Private acc.<br>☐2 Refugee camp<br>☐3 Institution[1]<br>☐4 Prison<br>☐5 Hospital<br>☐6 None, homeless<br>☐7 Hotel<br>☐8 Other: ____________<br>☐99 Don't know	☐99 Don't know
**13.5**	☐99 Don't know	☐1 Journey → *13c&d*<br>☐2 Stopover → *13e-g*<br>☐3 Other: ____________<br>☐99 Don't know	Destination A: ____________<br>Destination B: ____________<br>☐99 Don't know	☐1 Plane<br>☐2 Car, bus<br>☐3 Boat<br>☐4 By foot<br>☐5 Animal<br>☐6 Other: ____________<br>☐99 Don't know	Country: ____________<br>Ville: ____________<br>☐99 Don't know	☐1 Private acc.<br>☐2 Refugee camp<br>☐3 Institution[1]<br>☐4 Prison<br>☐5 Hospital<br>☐6 None, homeless<br>☐7 Hotel<br>☐8 Other: ____________<br>☐99 Don't know	☐99 Don't know

	a. Time period *(DD/MM/YY)*	b. Journey or stopover?	**Journey** c. Route *(Destination A – Destination B)*	d. Mode of transport	**Stopover** e. Place of stayover *(Country and town)*	f. Type of accommodation (acc.)	g. Name of accommodation, # of co-habitants
**13.6**	☐$_{99}$ Don't know	☐$_1$ Journey → *13c&d*<br>☐$_2$ Stopover → *13e-g*<br>☐$_3$ Other: ___________________<br>☐$_{99}$ Don't know	Destination A: __________________<br>Destination B: __________________<br>☐$_{99}$ Don't know	☐$_1$ Plane<br>☐$_2$ Car, bus<br>☐$_3$ Boat<br>☐$_4$ By foot<br>☐$_5$ Animal<br>☐$_6$ Other: ___________________<br>☐$_{99}$ Don't know	Country: __________________<br>Ville: __________________<br>☐$_{99}$ Don't know	☐$_1$ Private acc.<br>☐$_2$ Refugee camp<br>☐$_3$ Institution[1]<br>☐$_4$ Prison<br>☐$_5$ Hospital<br>☐$_6$ None, homeless<br>☐$_7$ Hotel<br>☐$_8$ Other: __________________<br>☐$_{99}$ Don't know	☐$_{99}$ Don't know
**13.7**	☐$_{99}$ Don't know	☐$_1$ Journey → *13c&d*<br>☐$_2$ Stopover → *13e-g*<br>☐$_3$ Other: ___________________<br>☐$_{99}$ Don't know	Destination A: __________________<br>Destination B: __________________<br>☐$_{99}$ Don't know	☐$_1$ Plane<br>☐$_2$ Car, bus<br>☐$_3$ Boat<br>☐$_4$ By foot<br>☐$_5$ Animal<br>☐$_6$ Other: ___________________<br>☐$_{99}$ Don't know	Country: __________________<br>Ville: __________________<br>☐$_{99}$ Don't know	☐$_1$ Private acc.<br>☐$_2$ Refugee camp<br>☐$_3$ Institution[1]<br>☐$_4$ Prison<br>☐$_5$ Hospital<br>☐$_6$ None, homeless<br>☐$_7$ Hotel<br>☐$_8$ Other: __________________<br>☐$_{99}$ Don't know	☐$_{99}$ Don't know
**13.8**	☐$_{99}$ Don't know	☐$_1$ Journey → *13c&d*<br>☐$_2$ Stopover → *13e-g*<br>☐$_3$ Other: ___________________<br>☐$_{99}$ Don't know	Destination A: __________________<br>Destination B: __________________<br>☐$_{99}$ Don't know	☐$_1$ Plane<br>☐$_2$ Car, bus<br>☐$_3$ Boat<br>☐$_4$ By foot<br>☐$_5$ Animal<br>☐$_6$ Other: ___________________<br>☐$_{99}$ Don't know	Country: __________________<br>Ville: __________________<br>☐$_{99}$ Don't know	☐$_1$ Private acc.<br>☐$_2$ Refugee camp<br>☐$_3$ Institution[1]<br>☐$_4$ Prison<br>☐$_5$ Hospital<br>☐$_6$ None, homeless<br>☐$_7$ Hotel<br>☐$_8$ Other: __________________<br>☐$_{99}$ Don't know	☐$_{99}$ Don't know

	a. Time period (DD/MM/YY)	b. Journey or stopover?	Journey: c. Route (Destination A – Destination B)	Journey: d. Mode of transport	Stopover: e. Place of stayover (Country and town)	Stopover: f. Type of accommodation (acc.)	Stopover: g. Name of accommodation, # of co-habitants
**13.9**	☐99 Don't know	☐1 Journey → *13c&d*<br>☐2 Stopover → *13e-g*<br>☐3 Other: ___________<br>☐99 Don't know	Destination A: ___________<br>Destination B: ___________<br>☐99 Don't know	☐1 Plane<br>☐2 Car, bus<br>☐3 Boat<br>☐4 By foot<br>☐5 Animal<br>☐6 Other: ___________<br>☐99 Don't know	Country: ___________<br>Ville: ___________<br>☐99 Don't know	☐1 Private acc.<br>☐2 Refugee camp<br>☐3 Institution[1]<br>☐4 Prison<br>☐5 Hospital<br>☐6 None, homeless<br>☐7 Hotel<br>☐8 Other: ___________<br>☐99 Don't know	☐99 Don't know
**13. 10**	☐99 Don't know	☐1 Journey → *13c&d*<br>☐2 Stopover → *13e-g*<br>☐3 Other: ___________<br>☐99 Don't know	Destination A: ___________<br>Destination B: ___________<br>☐99 Don't know	☐1 Plane<br>☐2 Car, bus<br>☐3 Boat<br>☐4 By foot<br>☐5 Animal<br>☐6 Other: ___________<br>☐99 Don't know	Country: ___________<br>Ville: ___________<br>☐99 Don't know	☐1 Private acc.<br>☐2 Refugee camp<br>☐3 Institution[1]<br>☐4 Prison<br>☐5 Hospital<br>☐6 None, homeless<br>☐7 Hotel<br>☐8 Other: ___________<br>☐99 Don't know	☐99 Don't know
**13. 11**	☐99 Don't know	☐1 Journey → *13c&d*<br>☐2 Stopover → *13e-g*<br>☐3 Other: ___________<br>☐99 Don't know	Destination A: ___________<br>Destination B: ___________<br>☐99 Don't know	☐1 Plane<br>☐2 Car, bus<br>☐3 Boat<br>☐4 By foot<br>☐5 Animal<br>☐6 Other: ___________<br>☐99 Don't know	Country: ___________<br>Ville: ___________<br>☐99 Don't know	☐1 Private acc.<br>☐2 Refugee camp<br>☐3 Institution[1]<br>☐4 Prison<br>☐5 Hospital<br>☐6 None, homeless<br>☐7 Hotel<br>☐8 Other: ___________<br>☐99 Don't know	☐99 Don't know

			Journey		Stopover		
	**a. Time period** *(DD/MM/YY)*	**b. Journey or stopover?**	**c. Route** *(Destination A – Destination B)*	**d. Mode of transport**	**e. Place of stayover** *(Country and town)*	**f. Type of accommodation (acc.)**	**g. Name of accommodation, # of co-habitants**
**13. 12**	☐$_{99}$ Don’t know	☐$_{1}$ Journey → *13c&d*<br>☐$_{2}$ Stopover → *13e-g*<br>☐$_{3}$ Other:<br>___________________<br>☐$_{99}$ Don’t know	Destination A:<br>__________________<br>Destination B:<br>__________________<br>☐$_{99}$ Don’t know	☐$_{1}$ Plane<br>☐$_{2}$ Car, bus<br>☐$_{3}$ Boat<br>☐$_{4}$ By foot<br>☐$_{5}$ Animal<br>☐$_{6}$ Other:<br>___________________<br>☐$_{99}$ Don’t know	Country:<br>__________________<br>Ville:<br>__________________<br>☐$_{99}$ Don’t know	☐$_{1}$ Private acc.<br>☐$_{2}$ Refugee camp<br>☐$_{3}$ Institution[1]<br>☐$_{4}$ Prison<br>☐$_{5}$ Hospital<br>☐$_{6}$ None, homeless<br>☐$_{7}$ Hotel<br>☐$_{8}$ Other:<br>__________________<br>☐$_{99}$ Don’t know	☐$_{99}$ Don’t know
**13. 13**	☐$_{99}$ Don’t know	☐$_{1}$ Journey → *13c&d*<br>☐$_{2}$ Stopover → *13e-g*<br>☐$_{3}$ Other:<br>___________________<br>☐$_{99}$ Don’t know	Destination A:<br>__________________<br>Destination B:<br>__________________<br>☐$_{99}$ Don’t know	☐$_{1}$ Plane<br>☐$_{2}$ Car, bus<br>☐$_{3}$ Boat<br>☐$_{4}$ By foot<br>☐$_{5}$ Animal<br>☐$_{6}$ Other:<br>___________________<br>☐$_{99}$ Don’t know	Country:<br>__________________<br>Ville:<br>__________________<br>☐$_{99}$ Don’t know	☐$_{1}$ Private acc.<br>☐$_{2}$ Refugee camp<br>☐$_{3}$ Institution[1]<br>☐$_{4}$ Prison<br>☐$_{5}$ Hospital<br>☐$_{6}$ None, homeless<br>☐$_{7}$ Hotel<br>☐$_{8}$ Other:<br>__________________<br>☐$_{99}$ Don’t know	☐$_{99}$ Don’t know
**13. 14**	☐$_{99}$ Don’t know	☐$_{1}$ Journey → *13c&d*<br>☐$_{2}$ Stopover → *13e-g*<br>☐$_{3}$ Other:<br>___________________<br>☐$_{99}$ Don’t know	Destination A:<br>__________________<br>Destination B:<br>__________________<br>☐$_{99}$ Don’t know	☐$_{1}$ Plane<br>☐$_{2}$ Car, bus<br>☐$_{3}$ Boat<br>☐$_{4}$ By foot<br>☐$_{5}$ Animal<br>☐$_{6}$ Other:<br>___________________<br>☐$_{99}$ Don’t know	Country:<br>__________________<br>Ville:<br>__________________<br>☐$_{99}$ Don’t know	☐$_{1}$ Private acc.<br>☐$_{2}$ Refugee camp<br>☐$_{3}$ Institution[1]<br>☐$_{4}$ Prison<br>☐$_{5}$ Hospital<br>☐$_{6}$ None, homeless<br>☐$_{7}$ Hotel<br>☐$_{8}$ Other:<br>__________________<br>☐$_{99}$ Don’t know	☐$_{99}$ Don’t know

**14**	Where did you, **for the first time**, enter Switzerland? *(as accurately as possible)*<br><br>☐99 Don't know / Refused	
**15**	When did you, **for the first time**, enter Switzerland? *(DD/MM/YYYY, as accurately as possible)*<br><br>☐99 Don't know / Refused	
**D. Information on the disease**		
**16a**	How and when did you notice your tuberculosis disease? When did the coughing begin, if ever? *(DD/MM/YYYY, as accurately as possible)*<br>____________________<br>____________________<br>____________________<br>☐1 Patient has noticed symptoms<br>☐2 Medical personnel at boarder control has noticed symptoms / TB<br>☐3 Examination with a medical doctor → *Go to 16b*<br>☐4 Was evaluated/examined because he/she had contact with another TB case<br>☐5 Has not noticed the disease<br>☐99 Don't know / Refused	*Ask as open-ended question. Check answers if applicable.*
**16b**	If answer 3 in Q16a: When, where and why were you at the doctors for a medical examination?<br>____________________<br>____________________<br>____________________	
**17a**	Did you know or suspect that you had tuberculosis **before** you arrived in Switzerland?<br>☐1 Yes → *17b*<br>☐2 No → *18*<br>☐99 Don't know / Refused → *18*	
**17b**	When was that? *(DD/MM/YYYY; as accurately as possible)*	

18 Did you experience the following symptoms of tuberculosis or have these symptoms increased during the progression of your tuberculosis disease? If yes, when was that?
Symptoms are also counted if they are recognized retrospectively, e.g. for coughing that was originally thought to be a persistent cold or smoker's cough.

		Yes	No	Don't know	DD/MM/YYYY *(as accurately as possible)*
**18.1**	Cough	☐1	☐2	☐99	
**18.2**	Cough with bloody sputum, haemoptysis	☐1	☐2	☐99	
**18.3**	Fever	☐1	☐2	☐99	
**18.4**	Weight loss	☐1	☐2	☐99	
**18.5**	Night sweats	☐1	☐2	☐99	
**18.6**	Fatigue	☐1	☐2	☐99	
**18.7**	Difficulties breathing, thorax/chest pain	☐1	☐2	☐99	
**18.8**	Others, please describe:<br>__________<br>__________<br>__________<br>__________	☐1	☐2	☐99	

**19a**	When did you, for the first time, seek medical advice or medical services (for example, a doctor or at a clinic) **outside Switzerland** because of **your tuberculosis symptoms / tuberculosis disease**? Please tell me as accurately as possible, the day and month and the year.	

	______________________________________<br>☐1 Never<br>☐99 Don't know / Refused	
**19b**	*If he sought medical help before entering Switzerland:* Where was this? What was the diagnosis, i.e. what did the medical staff tell you? Did you get medication? If so, which and for how long have you taken them?<br>____<br>____<br>____<br>____	
**19c**	When did you, for the first time, seek medical advice or medical services (for example, a doctor or at a clinic) **in Switzerland** because of **your tuberculosis symptoms / tuberculosis disease**? Please tell me as accurately as possible, the day and month and the year.<br>______________________________________<br>☐99 Don't know / Refused	
**20**	Where did this first medical advice or medical visit take place? *(Location and institution)*<br>____<br>____	
**E. Patient perception about the disease origin and contagion**		
**23**	In your opinion, where do you think you might have been infected with TB? Under what circumstances do you think that this infection has occurred? Why do you suspect that? Do you know if there were tuberculosis patients there? For example, did you live with a sick person in the same accommodation? Or did you travel with this person? How much time was spent with the person?<br>What is the name of this person, where is she from, how old, man or woman?<br>*(Country / City / Type and Name of Accommodation / Travel Section / Person)*<br>____<br>____<br>____<br>____<br>☐1 Appartment / house<br>☐2 Institution: Asylum seeker reception center; transit center, home for asylum seekers (house or apartment); homeless shelter<br>☐3 Prison<br>☐4 Hospital<br>☐5 Other(s): ____<br>☐99 Don't know / Refused	*Note: minimum duration of contact is 8h*
**24**	When and where did you last have contact with this person? We are talking about a personal contact for more than 8 hours, not a telephone or internet contact. *(DD/MM/YYYY and country / location, as accurately as possible.)*<br>____<br>____<br>☐99 Don't know / Refused	
**F. Other medical information**		
**25**	For the next question, I would like to remind you that you are free not to answer a question and that all your answers will be treated confidentially.<br>When did you last do a HIV test? *(MM/YYYY; as accurately as possible)*	

	____<br>☐1 Never<br>☐99 Don't know / Refused → *Gehe zu 27*	
**26**	Was the result of the HIV-test positive or negative?<br>☐1 Positive<br>☐2 Negative<br>☐99 Don't know / Refused	
**G. Closing questions and interview end**		
**27**	Do you have questions for us or do you want to tell us something else?<br>☐1 Yes:<br>____<br>____<br>☐2 No	
**28**	**Thank you for your participation!**	

Patient questionnaire – German/French version

Published with kind the permission of the Swiss Federal Office of Public Health.

Patienten-Fragebogen - Questionnaire avec le patient

A. Patienteninformation

A. Informations au patient

In der Schweiz wurden 2016 sieben Fälle von multiresistenter Lungentuberkulose diagnostiziert, die zu demselben Cluster gehören. Weitere Fälle vom selben Cluster wurden in anderen europäischen Länder diagnostiziert und deshalb wurde eine gesamteuropäische Investigation initiiert. In der Schweiz, wurde am 29. November 2016 das Kompetenzzentrum für Epidemiologische Ausbruchsuntersuchungen (KEA), geleitet vom Swiss Tropical and Public Health Institut (Swiss TPH), vom Schweizerischen Bundesamt für Gesundheit (BAG) mit einer epidemiologischen Untersuchung der Fälle beauftragt. Die Untersuchung erfolgt basierend auf Art. 20 der Meldeverordnung sowie auf Grundlage des Schweizerischen Epidemiengesetzes.

Da das Primärziel der Untersuchung die Ermittlung des/der Ansteckungsort(e) und/oder der Ansteckungswege ist, gilt es Herkunftsorte, Reiseroute und Aufenthaltsorte geographisch und zeitlich möglichst genau einzugrenzen.

Die Informationen die während dieses Interviews erhoben werden, werden streng vertraulich behandelt und sind nur den Studienmitarbeitern zugänglich. Insbesondere werden diese Informationen nicht den Migrationsbehörden zugestellt. Der Fragebogen wird in anonymisierter Form gehalten, wobei Ihnen eine Identifikationsnummer zugeteilt wird und Ihr Name nicht erwähnt oder notiert wird. Sie haben die Freiheit, Fragen nicht zu beantworten, ohne Angaben von Gründen.

En Suisse en 2016, huit cas de la tuberculose multirésistante (MDR-TB) ont été diagnostiqués, qui appartiennent au même ADN-Cluster. D'autres cas de du même Cluster ont été diagnostiqués dans d'autres pays Européens, ce qui a initié le lancement d'une enquête pan-européenne. En Suisse, le 29 Novembre 2016, le « Kompetenzzentrum für Epidemiologische Ausbruchsuntersuchungen (KEA) » (Centre de compétence pour les enquêtes épidémiologiques), dirigé par l'Institut Tropical et Santé Publique Suisse (Swiss TPH), a été chargée par l'Office Fédérale de Santé Publique Suisse (OFSP) de mener l'enquête épidémiologique de ces cas. L'enquête est effectuée sur la base de l'art. 20 du règlement de l'information et sur la base de la loi sur les épidémies en Suisse.

Puisque l'objectif principal de l'étude est de déterminer le(s) lieu(x) d'infection et/ou les voies d'infection, il est nécessaire de délimiter les lieux d'origine, l'itinéraire migratoire y inclue les séjours aux endroits données avec la plus grande précision possibles en termes de géographie et temporelle.

Les informations recueillies au cours de cet entretien seront strictement confidentielles et ne sont accessibles qu'au personnel de l'étude. En particulier, cette information ne sera pas partagée aux autorités de l'immigration. Le questionnaire sera conservé sous une forme anonyme. Un numéro d'identification vous sera attribué et votre nom ne sera pas mentionné ou cité dans le questionnaire. Vous avez la liberté de ne pas répondre à une question, sans donner de raisons.

**B. Informations générales**		**Comment.**
**1**	Numéro ID du patient:	
**2**	Sind Sie damit einverstanden, dass das Gespräch aufgenommen wird?<br>Êtes-vous d'accord que la conversation est enregistrée?<br>☐1 Oui<br>☐2 Non	
**3**	Sprachen des Interviews:<br>Langue(s) de l'entretien:<br>☐1 Allemand<br>☐2 Français<br>☐3 Italien<br>☐4 Anglais<br>☐5 Somalien<br>☐6 Tigrinya<br>☐7 Autre(s): ____________________________	
**4**	Übersetzung:<br>Traduction:<br>☐1 Non<br>☐2 Oui (spécifier): _________________________	

**5**	Personen die beim Interview präsent sind:<br>Personnes présentes à l'entretien:<br>☐1 Patient<br>☐2 Employé KEA<br>☐3 Emoloyé Ligue Pulmonaire<br>☐4 Traducteur<br>☐5 Autre: ________________________	
**6**	Geschlecht des Patienten:<br>Sexe du patient:<br>☐1 Homme<br>☐2 Femme	
**7**	Aktueller Wohn-Kanton:<br>Canton actuel de résidence:<br>☐1 St. Gall<br>☐2 Tessin<br>☐3 Vaud<br>☐4 Zürich	
**8**	Waren Sie vorher oder nachher in anderen Kantonen/Unterkünften in der Schweiz untergebracht?<br>Étiez-vous placé précédemment ou après dans d'autres cantons / hébergements en Suisse?<br>☐1 Oui<br>☐2 Non→ *10*<br>☐99 Weiss nicht, unbekannt→ *10*	*Siehe auf case listing Excel.*<br><br>*Voir case listing Excel.*
**9**	Wenn ja, wo und wann ?<br>Si oui, où et quand?	
**C. Demographische Informationen und Migrationsroute**<br>**C. Données démographiques et itinéraire migratoire**		
**10.1**	Geburtsdatum: *(JJ/MM/AAAA)*<br>Date de naissance : *(JJ/MM/AAAA)*<br>____________________________<br>☐99 Ne sait pas / Ne veut pas répondre	
**10.2**	Alter in Jahren:<br>Âge en années:<br>____________________________<br>☐99 Ne sait pas	
**11**	Können Sie uns genaue Angaben machen, wo Sie geboren wurden?<br>Pouvez-vous nous donner les détails de votre lieu de naissance ?<br><br>Land/Pays[1]: ____<br><br>Land/Pays[1]: ____<br><br>Ort/Lieu: ____	*[1]Le pays où la personne est née.*

	☐99 Ne sait pas / Ne veut pas répondre	
12	Können Sie die Zeitperioden und Wohnorte nennen während der letzten 2 Jahre bevor Sie ihr Land[2] verlassen haben?<br>Pouvez-vous nous énumérer les lieux et périodes de résidence des 2 dernières années avant de quitter votre pays?	[2]*Le pays où le voyage a commencé.*

**Période temporelle** ***(MM/JJ- MM/JJ)***	**Pays**	**Lieu : Ville / Village**

Diese Karten können mit den Fragen 13.0-13.14 benützt werden um die Migrationsroute zu rekonstruieren.

Celles cartes peuvent être utilisées avec les questions 13.0-13.14 pour reconstruire l'itinéraire migratoire.

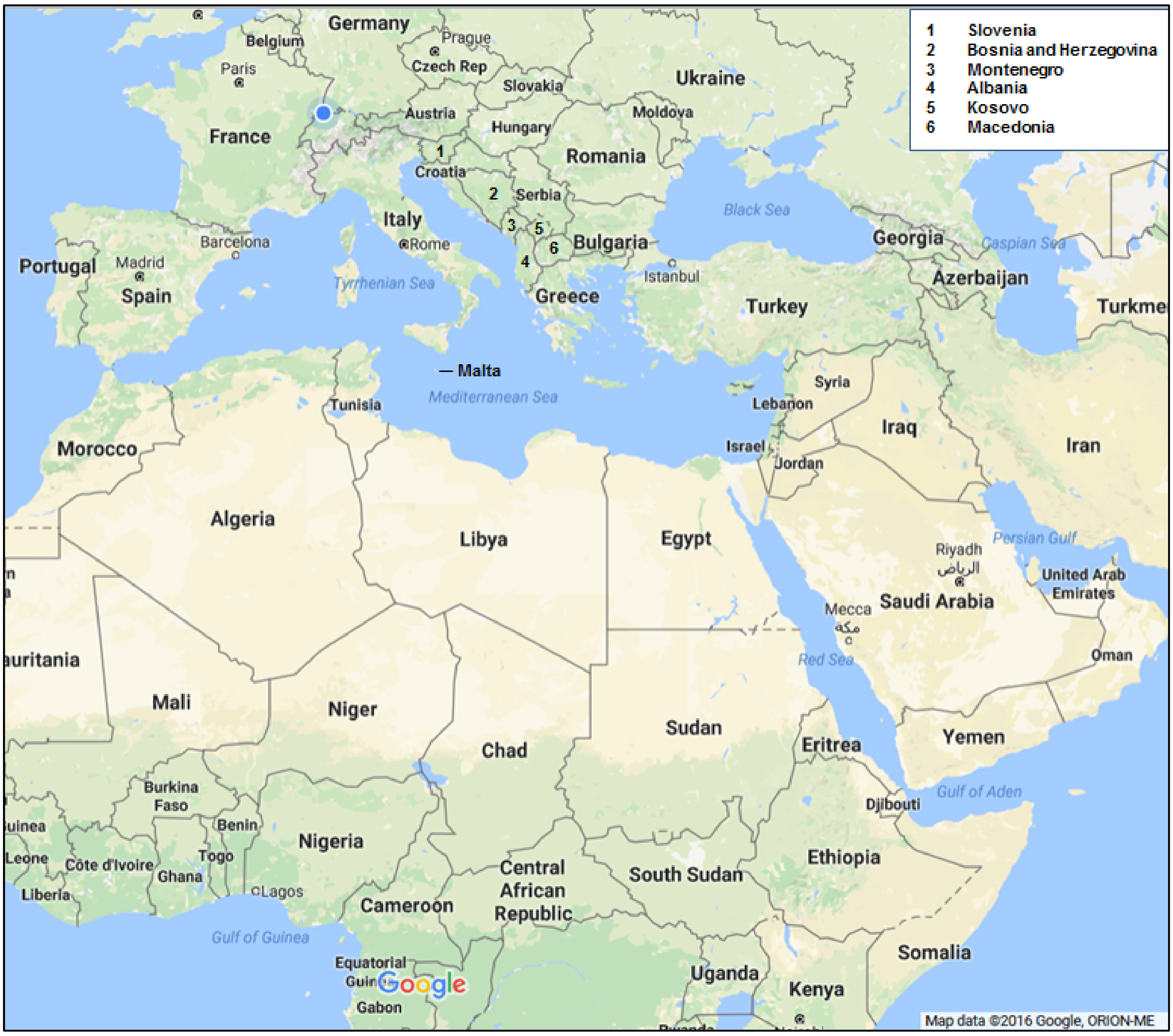

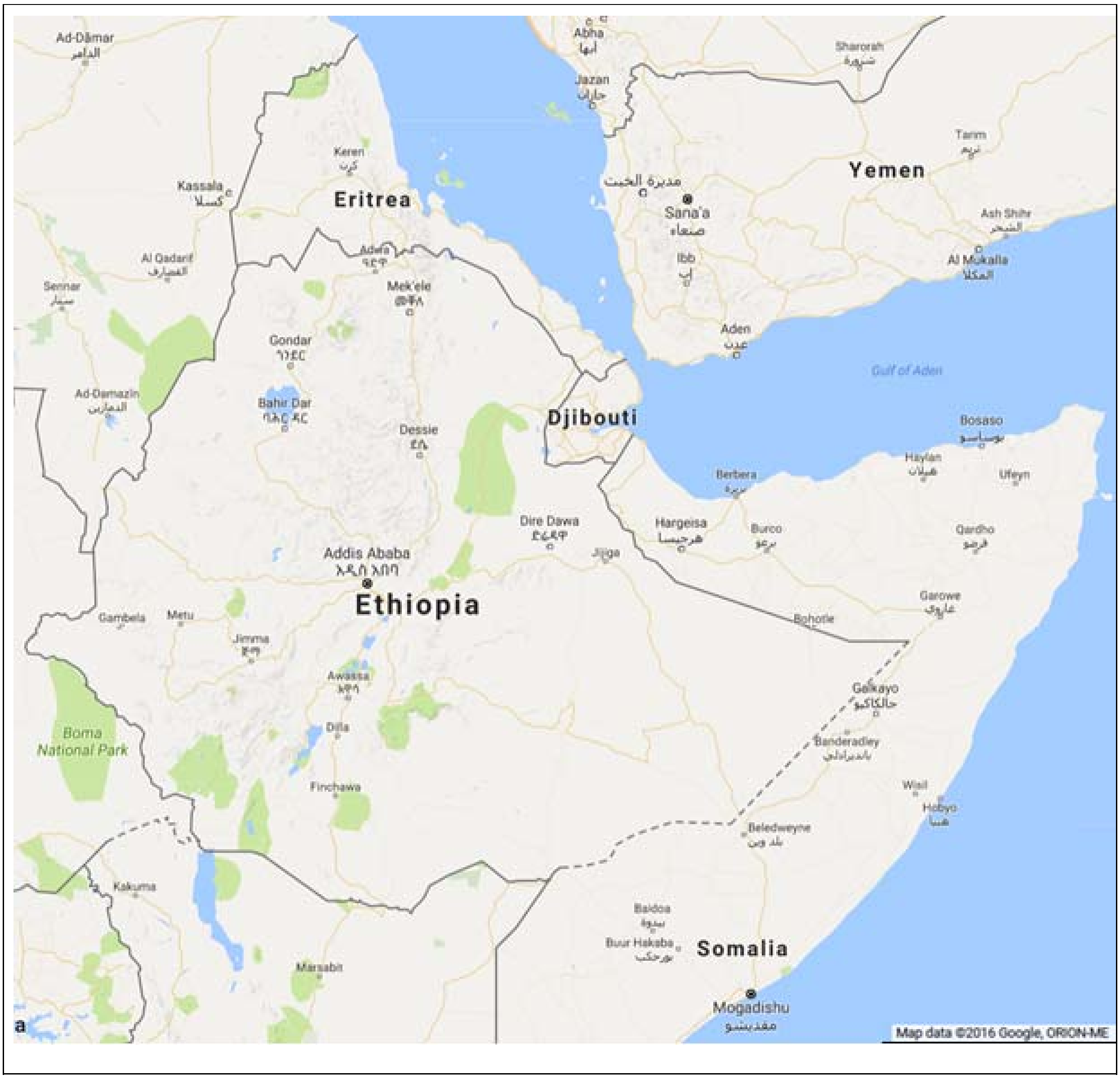
Ad-Dāmar
Abha
Jazan
Sharorah
Tarim
Yemen
Keren
Kassala
Eritrea
Sana'a
Ash Shihr
Adwa
Al Qadarif
Ibb
Al Mukalla
Sennar
Mek'ele
Gondar
Aden
Gulf of Aden
Ad-Damazin
Bahir Dar
Djibouti
Bosaso
Dessie
Haylan
Berbera
Ufeyn
Dire Dawa
Hargeisa
Burco
Qardho
Addis Ababa
Jijiga
Ethiopia
Garowe
Gambela
Metu
Bohotle
Jimma
Awassa
Galkayo
Boma
National Park
Dilla
Banderadley
Finchawa
Wisil
Hobyo
Beledweyne
Kakuma
Baidoa
Buur Hakaba
Somalia
Marsabit
Mogadishu
Map data ©2016 Google, ORION-ME

13	Wir wollen nun Ihre Migrationsroute chronologisch aufarbeiten. Sie können frei erzählen wie Ihre Migrationsroute verlief.<br>• Wenn möglich, bitte nennen Sie uns jeweils die Reiseetappen von einem Ort zu einem anderen Ort wie auch die Aufenthalte an einem Ort und die Zeitperioden.<br>• Wenn möglich, bitte nennen Sie uns auch die jeweiligen Transportmittel oder die Art der benutzen Unterkunft und der Name der Unterkunft, fall Sie sich daran erinnern.<br>Als Sie Ihre Migrationsreise in *„(letzter genannter Wohnort in Frage 12)“* gestartet, wann genau haben Sie diesen Ort verlassen *(JJ/MM/AAAA; so genau wie möglich)* und wie verlief ihre Reise dann?<br>Nous allons reconstruire la chronologie de votre itinéraire migratoire. Vous pouvez raconter de façon libre de votre itinéraire migratoire.<br>• Si possible, dites-nous les étapes distincts de **'voyage d'un endroit à un autre'** (déplacement) et les **séjours à un endroit donné** et la période temporelle correspondante.<br>• Si possible, dites-nous aussi le moyen de transport utilisé ou le type d'hébergement/logement et le nom de l'hébergement, si vous vous en souvenez.<br>Vous avez donc commencé votre voyage de migration à *"(le dernier lieu de résidence mentionné en question 12)"* ; quand avez-vous quitté cet endroit, exactement *(JJ/MM/AAAA; aussi précisément que possible)* et où êtes-vous allé ensuite?<br><br>*Hinweis: Fragen so weiterführen für jede Etappe. Jede Reise die durch einen Aufenthalt oder einen Wechsel des Transportmittel unterbrochen wurde, sollte als separate Etappe aufgelistet werden.*<br>*Note : Continuer les questions de la même manière pour chaque étape. Chaque voyage qui a été interrompu par un séjour ou un changement de moyen de transport doit être répertorié comme étape distincte.*<br>[1]*Institution [z.B. Empfangs- und Verfahrenszentrum (EVZ); Durchgangszentrum, Heim für Asylsuchende (Haus oder Wohnung); Obdachlosenheim]*<br>[1]*Institution [par ex. centre d'enregistrement et de procédure (CEP); centre de transit pour demandeurs d'asile, foyer pour requérants d'asile (maison ou appartement); centre d'hébergement pour sans-abri]*

			Voyage		Séjour		
	**a. Période** *(MM/AA-MM/AA)*	**b. Voyage ou séjour?**	**c. Route** *(Destination A – Destination B)*	**d. Moyen de transport**	**e. Lieu du séjour** *(Pays et ville)*	**f. Type d'hébergement (hb.)**	**g. Nom de l'hébergement**
**13.1**	☐99 Ne sait pas	☐1 Voyage → *13c&d*<br>☐2 Séjour → *13e-g*<br>☐3 Autre: ____________<br>☐99 Ne sait pas	Destination A: ____________<br>Destination B: ____________<br>☐99 Ne sait pas	☐1 Aéroplane<br>☐2 Voiture, bus<br>☐3 Bateau<br>☐4 À pied<br>☐5 Animale<br>☐6 Autre: ____________<br>☐99 Ne sait pas	Pays: ____________<br>Ville: ____________<br>☐99 Ne sait pas	☐1 hb. privé<br>☐2 Camp de réfugiés<br>☐3 Institution[1]<br>☐4 Prison<br>☐5 Hôpital<br>☐6 Sans abri<br>☐7 Hôtel<br>☐8 Autre: ____________<br>☐99 Ne sait pas	☐99 Ne sait pas
**13.2**		☐1 Voyage → *13c&d*<br>☐2 Séjour → *13e-g*<br>☐3 Autre: ____________	Destination A: ____________<br>Destination B: ____________	☐1 Aéroplane<br>☐2 Voiture, bus<br>☐3 Bateau<br>☐4 À pied<br>☐5 Animale	Pays: ____________<br>Ville: ____________	☐1 hb. privé<br>☐2 Camp de réfugiés<br>☐3 Institution[1]<br>☐4 Prison<br>☐5 Hôpital	

			Voyage		Séjour		
	**a. Période**<br>*(MM/AA-MM/AA)*	**b. Voyage ou séjour?**	**c. Route**<br>*(Destination A – Destination B)*	**d. Moyen de transport**	**e. Lieu du séjour**<br>*(Pays et ville)*	**f. Type d'hébergement (hb.)**	**g. Nom de l'hébergement**
	☐99 Ne sait pas	☐99 Ne sait pas	☐99 Ne sait pas	☐6 Autre: ____________<br>☐99 Ne sait pas	☐99 Ne sait pas	☐6 Sans abri<br>☐7 Hôtel<br>☐8 Autre: ____________<br>☐99 Ne sait pas	☐99 Ne sait pas
**13.3**	☐99 Ne sait pas	☐1 Voyage → *13c&d*<br>☐2 Séjour → *13e-g*<br>☐3 Autre: ____________<br>☐99 Ne sait pas	Destination A: ____________<br>Destination B: ____________<br>☐99 Ne sait pas	☐1 Aéroplane<br>☐2 Voiture, bus<br>☐3 Bateau<br>☐4 À pied<br>☐5 Animale<br>☐6 Autre: ____________<br>☐99 Ne sait pas	Pays: ____________<br>Ville: ____________<br>☐99 Ne sait pas	☐1 hb. privé<br>☐2 Camp de réfugiés<br>☐3 Institution[1]<br>☐4 Prison<br>☐5 Hôpital<br>☐6 Sans abri<br>☐7 Hôtel<br>☐8 Autre: ____________<br>☐99 Ne sait pas	☐99 Ne sait pas
**13.4**	☐99 Ne sait pas	☐1 Voyage → *13c&d*<br>☐2 Séjour → *13e-g*<br>☐3 Autre: ____________<br>☐99 Ne sait pas	Destination A: ____________<br>Destination B: ____________<br>☐99 Ne sait pas	☐1 Aéroplane<br>☐2 Voiture, bus<br>☐3 Bateau<br>☐4 À pied<br>☐5 Animale<br>☐6 Autre: ____________<br>☐99 Ne sait pas	Pays: ____________<br>Ville: ____________<br>☐99 Ne sait pas	☐1 hb. privé<br>☐2 Camp de réfugiés<br>☐3 Institution[1]<br>☐4 Prison<br>☐5 Hôpital<br>☐6 Sans abri<br>☐7 Hôtel<br>☐8 Autre: ____________<br>☐99 Ne sait pas	☐99 Ne sait pas
**13.5**		☐1 Voyage → *13c&d*<br>☐2 Séjour → *13e-g*<br>☐3 Autre: ____________	Destination A: ____________<br>Destination B: ____________	☐1 Aéroplane<br>☐2 Voiture, bus<br>☐3 Bateau<br>☐4 À pied<br>☐5 Animale<br>☐6 Autre: ____________	Pays: ____________<br>Ville: ____________	☐1 hb. privé<br>☐2 Camp de réfugiés<br>☐3 Institution[1]<br>☐4 Prison<br>☐5 Hôpital<br>☐6 Sans abri<br>☐7 Hôtel<br>☐8 Autre: ____________	

	Voyage				Séjour		
	**a. Période** *(MM/AA-MM/AA)*	**b. Voyage ou séjour?**	**c. Route** *(Destination A – Destination B)*	**d. Moyen de transport**	**e. Lieu du séjour** *(Pays et ville)*	**f. Type d'hébergement (hb.)**	**g. Nom de l'hébergement**
	☐99 Ne sait pas	☐99 Ne sait pas	☐99 Ne sait pas	☐99 Ne sait pas	☐99 Ne sait pas	☐99 Ne sait pas	☐99 Ne sait pas
**13.6**	☐99 Ne sait pas	☐1 Voyage → *13c&d*<br>☐2 Séjour → *13e-g*<br>☐3 Autre: ___________<br>☐99 Ne sait pas	Destination A: ___________<br>Destination B: ___________<br>☐99 Ne sait pas	☐1 Aéroplane<br>☐2 Voiture, bus<br>☐3 Bateau<br>☐4 À pied<br>☐5 Animale<br>☐6 Autre: ___________<br>☐99 Ne sait pas	Pays: ___________<br>Ville: ___________<br>☐99 Ne sait pas	☐1 hb. privé<br>☐2 Camp de réfugiés<br>☐3 Institution[1]<br>☐4 Prison<br>☐5 Hôpital<br>☐6 Sans abri<br>☐7 Hôtel<br>☐8 Autre: ___________<br>☐99 Ne sait pas	☐99 Ne sait pas
**13.7**	☐99 Ne sait pas	☐1 Voyage → *13c&d*<br>☐2 Séjour → *13e-g*<br>☐3 Autre: ___________<br>☐99 Ne sait pas	Destination A: ___________<br>Destination B: ___________<br>☐99 Ne sait pas	☐1 Aéroplane<br>☐2 Voiture, bus<br>☐3 Bateau<br>☐4 À pied<br>☐5 Animale<br>☐6 Autre: ___________<br>☐99 Ne sait pas	Pays: ___________<br>Ville: ___________<br>☐99 Ne sait pas	☐1 hb. privé<br>☐2 Camp de réfugiés<br>☐3 Institution[1]<br>☐4 Prison<br>☐5 Hôpital<br>☐6 Sans abri<br>☐7 Hôtel<br>☐8 Autre: ___________<br>☐99 Ne sait pas	☐99 Ne sait pas
**13.8**	☐99 Ne sait pas	☐1 Voyage → *13c&d*<br>☐2 Séjour → *13e-g*<br>☐3 Autre: ___________<br>☐99 Ne sait pas	Destination A: ___________<br>Destination B: ___________<br>☐99 Ne sait pas	☐1 Aéroplane<br>☐2 Voiture, bus<br>☐3 Bateau<br>☐4 À pied<br>☐5 Animale<br>☐6 Autre: ___________<br>☐99 Ne sait pas	Pays: ___________<br>Ville: ___________<br>☐99 Ne sait pas	☐1 hb. privé<br>☐2 Camp de réfugiés<br>☐3 Institution[1]<br>☐4 Prison<br>☐5 Hôpital<br>☐6 Sans abri<br>☐7 Hôtel<br>☐8 Autre: ___________<br>☐99 Ne sait pas	☐99 Ne sait pas
**13.9**		☐1 Voyage → *13c&d*<br>☐2 Séjour → *13e-g*<br>☐3 Autre:	Destination A: ___________	☐1 Aéroplane<br>☐2 Voiture, bus<br>☐3 Bateau	Pays: ___________	☐1 hb. privé<br>☐2 Camp de réfugiés<br>☐3 Institution[1]	

	a. Période (MM/AA-MM/AA)	b. Voyage ou séjour?	Voyage: c. Route (Destination A – Destination B)	Voyage: d. Moyen de transport	Séjour: e. Lieu du séjour (Pays et ville)	Séjour: f. Type d'hébergement (hb.)	Séjour: g. Nom de l'hébergement
	☐99 Ne sait pas	____________<br>☐99 Ne sait pas	Destination B: ____________<br>☐99 Ne sait pas	☐4 À pied<br>☐5 Animale<br>☐6 Autre: ____________<br>☐99 Ne sait pas	Ville: ____________<br>☐99 Ne sait pas	☐4 Prison<br>☐5 Hôpital<br>☐6 Sans abri<br>☐7 Hôtel<br>☐8 Autre: ____________<br>☐99 Ne sait pas	☐99 Ne sait pas
**13.10**	☐99 Ne sait pas	☐1 Voyage → *13c&d*<br>☐2 Séjour → *13e-g*<br>☐3 Autre: ____________<br>☐99 Ne sait pas	Destination A: ____________<br>Destination B: ____________<br>☐99 Ne sait pas	☐1 Aéroplane<br>☐2 Voiture, bus<br>☐3 Bateau<br>☐4 À pied<br>☐5 Animale<br>☐6 Autre: ____________<br>☐99 Ne sait pas	Pays: ____________<br>Ville: ____________<br>☐99 Ne sait pas	☐1 hb. privé<br>☐2 Camp de réfugiés<br>☐3 Institution[1]<br>☐4 Prison<br>☐5 Hôpital<br>☐6 Sans abri<br>☐7 Hôtel<br>☐8 Autre: ____________<br>☐99 Ne sait pas	☐99 Ne sait pas
**13.11**	☐99 Ne sait pas	☐1 Voyage → *13c&d*<br>☐2 Séjour → *13e-g*<br>☐3 Autre: ____________<br>☐99 Ne sait pas	Destination A: ____________<br>Destination B: ____________<br>☐99 Ne sait pas	☐1 Aéroplane<br>☐2 Voiture, bus<br>☐3 Bateau<br>☐4 À pied<br>☐5 Animale<br>☐6 Autre: ____________<br>☐99 Ne sait pas	Pays: ____________<br>Ville: ____________<br>☐99 Ne sait pas	☐1 hb. privé<br>☐2 Camp de réfugiés<br>☐3 Institution[1]<br>☐4 Prison<br>☐5 Hôpital<br>☐6 Sans abri<br>☐7 Hôtel<br>☐8 Autre: ____________<br>☐99 Ne sait pas	☐99 Ne sait pas
**13.12**		☐1 Voyage → *13c&d*<br>☐2 Séjour → *13e-g*<br>☐3 Autre: ____________	Destination A: ____________<br>Destination B: ____________	☐1 Aéroplane<br>☐2 Voiture, bus<br>☐3 Bateau<br>☐4 À pied<br>☐5 Animale<br>☐6 Autre: ____________	Pays: ____________<br>Ville: ____________	☐1 hb. privé<br>☐2 Camp de réfugiés<br>☐3 Institution[1]<br>☐4 Prison<br>☐5 Hôpital<br>☐6 Sans abri<br>☐7 Hôtel	

	a. Période *(MM/AA-MM/AA)*	b. Voyage ou séjour?	Voyage: c. Route *(Destination A – Destination B)*	Voyage: d. Moyen de transport	Séjour: e. Lieu du séjour *(Pays et ville)*	Séjour: f. Type d'hébergement (hb.)	Séjour: g. Nom de l'hébergement
	☐99 Ne sait pas	☐99 Ne sait pas	☐99 Ne sait pas	☐99 Ne sait pas	☐99 Ne sait pas	☐8 Autre: ____________<br>☐99 Ne sait pas	☐99 Ne sait pas
**13. 13**	☐99 Ne sait pas	☐1 Voyage → *13c&d*<br>☐2 Séjour → *13e-g*<br>☐3 Autre: ____________<br>☐99 Ne sait pas	Destination A: ____________<br>Destination B: ____________<br>☐99 Ne sait pas	☐1 Aéroplane<br>☐2 Voiture, bus<br>☐3 Bateau<br>☐4 À pied<br>☐5 Animale<br>☐6 Autre: ____________<br>☐99 Ne sait pas	Pays: ____________<br>Ville: ____________<br>☐99 Ne sait pas	☐1 hb. privé<br>☐2 Camp de réfugiés<br>☐3 Institution[1]<br>☐4 Prison<br>☐5 Hôpital<br>☐6 Sans abri<br>☐7 Hôtel<br>☐8 Autre: ____________<br>☐99 Ne sait pas	☐99 Ne sait pas
**13. 14**	☐99 Ne sait pas	☐1 Voyage → *13c&d*<br>☐2 Séjour → *13e-g*<br>☐3 Autre: ____________<br>☐99 Ne sait pas	Destination A: ____________<br>Destination B: ____________<br>☐99 Ne sait pas	☐1 Aéroplane<br>☐2 Voiture, bus<br>☐3 Bateau<br>☐4 À pied<br>☐5 Animale<br>☐6 Autre: ____________<br>☐99 Ne sait pas	Pays: ____________<br>Ville: ____________<br>☐99 Ne sait pas	☐1 hb. privé<br>☐2 Camp de réfugiés<br>☐3 Institution[1]<br>☐4 Prison<br>☐5 Hôpital<br>☐6 Sans abri<br>☐7 Hôtel<br>☐8 Autre: ____________<br>☐99 Ne sait pas	☐99 Ne sait pas

**14**	Wo sind Sie (das erste Mal) in die Schweiz eingereist? *(so genau wie möglich)*<br>Par où êtes-vous entré en Suisse pour **la première fois**? *(Aussi précisément que possible)*	
**15**	Wann sind Sie **das erste Mal** in die Schweiz eingereist? *(TT/MM/JJJJ; so genau wie möglich)*<br>Quand êtes-vous entré en Suisse pour **la première fois**? *(JJ/MM/AAAA ; Aussi précisément que possible)*<br><br>☐99 Ne sait pas / Ne veut pas répondre	
**D. Informationen zur Tuberkulose-Erkrankung**<br>**D. Informations sur la maladie de la tuberculose**		
**16a**	Wie und wann haben Sie ihre Tuberkulose-Erkrankung bemerkt? Wann begann der Husten, falls je gehabt? *(TT/MM/JJJJ; so genau wie möglich)*<br>Comment et quand vous avez remarqué votre infection par la tuberculose? Si vous avez jamais eu la toux, quand est-ce que la toux a commencée? *(JJ/MM/AAAA ; Aussi précisément que possible)*<br>____<br>____<br>____<br>☐1 Patient hat Symptome bemerkt<br>☐1 Le patient a constaté les symptômes<br>☐2 Medizinisches Personal/Grenzsanität hat die TB bemerkt<br>☐2 Le personnel médical / le service sanitaire de frontière a constaté la tuberculose<br>☐3 Untersuchung beim Arzt → *Gehe zu 16b*<br>☐3 Lors d'un examen chez le médecin → *16b*<br>☐4 Wurde untersucht, da ich Kontakt mit einem anderen TB Patienten hatte<br>☐4 A été examiné suite à un contact avec un autre patient tuberculeux<br>☐5 Hat die Krankheit nicht bemerkt<br>☐5 N'a pas constaté la maladie<br>☐99 Weiss nicht / Will nicht antworten<br>☐99 Ne sait pas / Ne veut pas répondre	*Frage offen stellen. Geschlossene Angaben ankreuzen je nach Relevanz.*<br><br>*Question ouverte. Checkbox en fonction de leur pertinence.*
**16b**	Falls Antwort 3 in Q16a: Wann, wo und warum waren Sie beim Arzt für eine Untersuchung?<br>Si réponse 3 en Q16a: Quand, où et pourquoi vous étiez chez le médecin pour un examen?<br>____<br>____<br>____	
**17a**	**Ahnten** oder wussten Sie, dass Sie an Tuberkulose erkrankt sind **bevor Sie in die Schweiz einreisten**?<br>Avez-vous suspecté ou avez-vous su que vous souffriez de tuberculose **avant d'entrer en Suisse**?<br>☐1 Oui → *17b*<br>☐2 Non → *18*<br>☐99 Ne sait pas / Ne veut pas répondre → *18*	
**17b**	Seit wann? ? *(TT/MM/JJJJ; so genau wie möglich)*<br>Depuis quand? *(JJ/MM/AAAA ; Aussi précisément que possible)*	
**18**	Sind folgende Symptome im Rahmen der Tuberkuloseerkrankung aufgetreten oder haben diese Symptome durch die Tuberkulose- erkrankung zugenommen? Wenn Ja, wann war das?<br>Les symptômes suivants sont-ils apparus ou ont-ils augmenté dans le contexte de la tuberculose? Si oui, quand était-ce?<br>*Symptome sind auch zu zählen wenn sie erst retrospektiv erkannt werden, z.B. für Husten der ursprünglich für eine anhaltende Erkältung oder Raucherhusten gehalten wurde.*<br>*Les symptômes sont également à compter quand ils ne sont reconnus que rétrospectivement, comme par exemple pour une toux qui a été initialement considérée comme une toux froide ou toux du fumeur.*	

	Oui	Non	Ne sait pas	JJ/MM/AAAA *(so genau wie möglich)*

**18.1**	Husten<br>Toux	☐1	☐2	☐99	
**18.2**	Bluthusten<br>Toux sanglante	☐1	☐2	☐99	
**18.3**	Fieber<br>Fièvre	☐1	☐2	☐99	
**18.4**	Gewichtsabnahme<br>Perte de poids	☐1	☐2	☐99	
**18.5**	Vermehrtes Schwitzen (besonders nachts)<br>Transpiration accrue (surtout la nuit)	☐1	☐2	☐99	
**18.6**	Müdigkeit und/oder Abgeschlagenheit<br>Fatigue et/ou abattement	☐1	☐2	☐99	
**18.7**	Brustschmerzen und/oder Atemnot<br>Douleurs thoraciques et/ou essoufflement	☐1	☐2	☐99	
**18.8**	Sonstige, bitte nennen:<br>Autres, veuillez préciser:<br>____<br>____<br>____<br>____	☐1	☐2	☐99	
**19a**	Wann haben Sie zum ersten Mal **wegen Ihrer Symptome/Tuberkuloseerkrankung** medizinische Dienste (z.B. einen Arzt/eine Ärztin oder eine Klinik) **ausserhalb der Schweiz** beansprucht? Bitte sagen Sie mir dies möglichst genau, Tag und Monat und Jahr.<br>Quand avez-vous consulté les services médicaux (par exemple un médecin ou une clinique) pour la première fois **au dehors de la Suisse à cause de vos symptômes / de la tuberculose**? SVP me dire cela aussi précisément que possible, jour, mois et année.<br><br>______________________________________<br>☐1 Jamais<br>☐99 Ne sait pas / Ne veut pas répondre				
**19b**	*Falls er vor Einreise in die Schweiz medizinische Hilfe beansprucht hat:* Wo war dies? Was war die Diagnose, d.h. was hat Ihnen das medizinische Personal gesagt? Haben Sie Medikamente bekommen? Falls ja, welche und für wie lange haben Sie diese genommen?<br>*S'il a consulté des services médicaux avant l'entrée en Suisse:* Où était-ce? Quel était le diagnose, c'est-à-dire qu'est-ce que le personnel médical vous a dit? Avez-vous reçu des médicaments/comprimées? Si oui, quoi et pour combien de temps vous avez pris ces comprimés?<br>____<br>____<br>____<br>____<br>____<br>____<br>____				
**19c**	Wann haben Sie zum ersten Mal **wegen Ihrer Symptome/Tuberkuloseerkrankung** medizinische Dienste (z.B. einen Arzt/eine Ärztin oder eine Klinik) **in der Schweiz** beansprucht? Bitte sagen Sie mir dies möglichst genau, Tag und Monat und Jahr. *(JJ/MM/AAAA; so genau wie möglich)*<br>Quand avez-vous consulté les services médicaux (par exemple un médecin ou une clinique) pour la première **en Suisse fois à cause de vos symptômes / de la tuberculose**? SVP me dire cela aussi précisément que possible, jour, mois et année.<br><br>______________________________________<br>☐99 Ne sait pas / Ne veut pas répondre				
**20**	Wo fand diese erste (Arzt)-Konsultation in der Schweiz statt? *(Ort und Struktur)*<br>Dans quelle structure cette première consultation a eu lieu en Suisse? *(Lieu et structure)*<br>____<br>____				

	______________________________<br>______________________________<br>______________________________	
**E. Patientenwahrnehmung über die Krankheitsherkunft und –ansteckung**<br>**E. La perception du patient concernant les origines et l'infection par la maladie**		
**23**	Ihrer Meinung nach, wo denken Sie, könnten Sie mit TB infiziert worden sein? Unter welchen Umständen meinen Sie hat eine Infektion stattgefunden? Warum meinen Sie das? Wissen Sie ob es dort Tuberkulose Kranke gab? Zum Beispiel, haben Sie mit einer erkrankten Person in der gleichen Unterkunft gelebt? Oder sind Sie mit dieser Person gereist? Wie viel Zeit wurde mit der Person verbracht?<br>Wie heisst diese Person, woher ist sie, etwa wie alt, Mann oder Frau?<br>*(Land / Ort / Typ und Name der Unterkunft / Reiseabschnitt / Person)*<br>À votre avis, où pensez-vous que vous pourriez avoir été infecté par la tuberculose (A votre avis, où pourriez avoir contracté la tuberculose)? Dans quelles circonstances pensez-vous que l'infection a pu avoir lieu? Pourquoi pensez-vous cela ? Savez-vous s'il y avait des patients atteints de tuberculose à cet endroit? Avez-vous vécu dans le même logement qu'une personne infectée, par exemple? Ou avez-vous voyagé avec cette personne? Combien de temps avez-vous passé avec cette personne?<br>Quel est le nom de cette personne, où est-il maintenant, quel âge, masculin ou féminin?<br>*(Pays / Région / Nom de l'hébergement / étape de voyage / personne)*<br>______________________________<br>______________________________<br>______________________________<br>______________________________<br>______________________________<br>______________________________<br>☐1 Wohnung / Haus (gemietet oder Eigentum)<br>☐1 Appartement ou maison (loué/e par vous-même ou en propriété)<br>☐2 Institution [z.B. Empfangs- und Verfahrenszentrum (EVZ); Durchgangszentrum, Heim für Asylsuchende (Haus oder Wohnung); Obdachlosenheim]<br>☐2 Institution [par ex. centre d'enregistrement et de procédure (CEP); centre de transit pour demandeurs d'asile, foyer pour requérants d'asile (maison ou appartement); centre d'hébergement pour sans-abri]<br>☐3 Haftanstalt, Gefängnis<br>☐3 Prison<br>☐4 Spital<br>☐4 Hôpital<br>☐5 Andere: ______________________<br>☐5 Autre(s): ______________________<br>☐99 Weiss nicht / Will nicht antworten<br>☐99 Ne sait pas / Ne veut pas répondre	*Hinweis: mindestens 8 Std.*<br><br>*Frage nach potentieller Person*<br><br>*Indication: au moins 8 heures.*<br><br>*Question relative à personne potentielle*
**24**	Wann und wo hatten sie das letzte Mal Kontakt mit dieser möglichen Person? Wir sprechen hier von einem persönlichen Kontakt über mehr als 8 Stunden, nicht einen Telefon- oder Internetkontakt. *(TT/MM/JJJ und Land/Ort; so genau wie möglich.)*<br>Quand et où avez-vous eu le dernier contact avec cette personne? Nous parlons d'un contact en personne de plus de 8 heures et non d'un contact téléphonique ou par le net. *(JJ / MM / AAAA et le pays / place; aussi précisément que possible.)*<br>______________________________<br>______________________________<br>______________________________	

	☐$_{99}$ Ne sait pas / Ne veut pas répondre	
**F. Andere medizinische Angaben**<br>**F. Autres informations médicaux**		
**25**	Für die nächste Frage möchte ich Sie daran erinnern, dass Sie frei sind, eine Frage nicht zu beantworten und dass alle Ihre Antworten vertraulich behandelt werden.<br>Wann haben Sie ihren letzten HIV-Test gemacht? *(MM/JJJJ; so genau wie möglich)*<br>Pour la prochaine question, je voudrais vous rappeler que vous êtes libre de ne pas répondre à une question et que toutes vos réponses sont confidentielles.<br>Quand avez-vous fait votre dernier test de VIH? (MM/AAAA, aussi précisément que possible)<br><br>____<br>☐$_{1}$ Nie<br>☐$_{99}$ Weiss nicht / Will nicht antworten → *Gehe zu 27*	
**26**	War der HIV-Test positiv oder negativ?<br>Le test VIH était positif ou négatif?<br>☐$_{1}$ Positif<br>☐$_{2}$ Négatif<br>☐$_{99}$ Ne sait pas / Ne veut pas répondre	
**G. Abschliessende Fragen und Ende des Interviews**<br>**G. Questions finales et fin de l'entretien**		
**27**	Haben Sie noch irgendwelche Fragen oder möchten Sie uns etwas mitteilen?<br>Avez-vous des questions ou voulez-vous nous dire quelque chose?<br>☐$_{1}$ Oui:<br>____<br><br>____<br>☐$_{2}$ Non<br>☐$_{99}$ Ne sait pas / Ne veut pas répondre	
**28**	**Vielen Dank für Ihre Teilnahme.**<br>**Nous vous remercions chaleureusement pour votre participation.**	

Supplementary Table 1. Main characteristics of the 29 outlier patients and susceptibility testing result of the outlier MDR-TB isolates.

Patient identifier	Gender*	Child (< 18 yrs.)/ adult	Country of diagnosis	Country where journey started	Month of diagnosis‡	Site of disease	Microscopy result¶	Fastq accession number	Phenotypic drug susceptibility testing results (critical concentration testing)§												
									INH	RIF	EMB	PZA	SM	MOX	OFL	AMI	KAN	CAP	ETH	PAS	LIN
Belgium 1	F	Adult	Belgium	Djibouti	Nov. 2013	Lymph nodes	Unknown	ERS1927713	R	R	S	R	nd	S	S	S	nd	R	S	S	S
Belgium 2	F	Adult	Belgium	Somalia	Aug. 2011	Pulmonary	Positive	ERS1927714	R	R	S	R	nd	S	S	S	nd	R	S	R	S
Djibouti 1	na	na	Djibouti	na	na	na	na	ERS1927729	S	S	S	S	nd	nd	nd	nd	nd	R	nd	nd	nd
Djibouti 2	na	na	Djibouti	na	na	na	na	ERS1927730	S	S	S	S	nd	nd	nd	nd	nd	R	nd	nd	nd
Djibouti 3	na	na	Djibouti	na	na	na	na	ERS1927731	S	S	S	S	nd	nd	nd	nd	nd	R	nd	nd	nd
Djibouti 4	na	na	Djibouti	na	na	na	na	ERS1927732	S	S	S	S	nd	nd	nd	nd	nd	R	nd	nd	nd
Djibouti 5	na	na	Djibouti	na	na	na	na	ERS1927733	S	S	S	S	nd	nd	nd	nd	nd	R	nd	nd	nd
Djibouti 6	na	na	Djibouti	na	na	na	na	ERS1927734	S	S	S	S	nd	nd	nd	nd	nd	R	nd	nd	nd
Finland 1	na	na	Finland	na	na	na	na	ERS1927736	S	S	S	S	nd	nd	nd	nd	nd	nd	nd	nd	nd
Finland 2	na	na	Finland	na	na	na	na	ERS1927737	S	S	S	S	nd	nd	nd	nd	nd	nd	nd	nd	nd
Finland 3	na	na	Finland	na	na	na	na	ERS1927738	S	S	S	S	nd	nd	nd	nd	nd	nd	nd	nd	nd
Ireland 1	na	na	Ireland	na	na	na	na	ERS1927741	S	S	S	S	nd	nd	nd	nd	nd	R	nd	nd	nd
Italy 1	na	na	Italy	na	na	na	na	ERS1927742	S	na	na	na	nd	nd	nd	nd	nd	R	nd	nd	nd
Italy 2	na	na	Italy	na	na	na	na	ERS1927743	R	na	na	na	nd	nd	nd	nd	nd	R	nd	nd	nd
Italy 3	na	na	Italy	na	na	na	na	ERS1927744	R	na	na	na	nd	nd	nd	nd	nd	R	nd	nd	nd
Netherlands 1	na	na	Netherlands	na	na	na	na	ERS1927745	S	S	S	S	nd	nd	nd	nd	nd	nd	nd	nd	nd
Netherlands 2	na	na	Netherlands	na	na	na	na	ERS1927746	S	S	S	S	nd	nd	nd	nd	nd	nd	nd	nd	nd
Netherlands 3	na	na	Netherlands	na	na	na	na	ERS1927747	R	R	S	S	S	S	nd	S	S	R	nd	nd	S

Norway 1	na	Adult	Norway	Somalia	na	Pulmonary	na	ERS1927748	R	R	R	R	R	nd	S	S	R	R	U	U	S
Norway 2	na	Adult	Norway	Ethiopia	na	Extra-pulmonary	na	ERS1927749	S	S	S	S	S	nd	nd	nd	nd	S	nd	nd	nd
Norway 3	na	Adult	Norway	Ethiopia	na	Extra-pulmonary	na	ERS1927750	S	S	S	S	S	nd	nd	nd	nd	S	nd	nd	nd
UK 2	na	Adult	UK	na	na	na	na	ERS1927760	S	S	S	S	nd	nd	nd	nd	nd	nd	nd	nd	nd
UK 3	na	Adult	UK	na	na	na	na	ERS1927761	S	S	S	S	nd	nd	nd	nd	nd	nd	nd	nd	nd
UK 4	na	Adult	UK	na	na	na	na	ERS1927762	S	S	S	S	nd	nd	nd	nd	nd	nd	nd	nd	nd
UK 5	na	Adult	UK	na	na	na	na	ERS1927763	S	S	S	S	nd	nd	nd	nd	nd	nd	nd	nd	nd
UK 6	na	Adult	UK	na	na	na	na	ERS1927764	S	S	S	S	nd	nd	nd	nd	nd	nd	nd	nd	nd
UK 7	na	Adult	UK	na	na	na	na	ERS1927765	S	S	S	S	nd	nd	nd	nd	nd	nd	nd	nd	nd
UK 8	na	Adult	UK	na	na	na	na	ERS1927766	S	S	S	S	nd	nd	nd	nd	nd	nd	nd	nd	nd
UK 9	na	Adult	UK	na	na	na	na	ERS1927767	S	S	S	S	nd	nd	nd	nd	nd	nd	nd	nd	nd

* Abbreviations: na – not available, M – male, F – female. ‡ According to the state where the patient was first diagnosed. ¶ Qualitative result of fluorescence direct material microscopy (auramine-rhodamine staining). § Abbreviations: INH – isoniazid, RIF – rifampicin, EMB – ethambutol, PZA – pyrazinamide (of note: all outbreak isolates had the *pncA*_W68C mutation), SM – streptomycin, MOX – moxifloxacin, OFL – ofloxacin, AMI – amikazin, KAN – kanamycin A, CAP – capreomycin, ETH – ethionamide, PTH – prothionamide, CS – cycloserine, PAS – para-aminosalicylic acid, BDQ – bedaquiline, DLM – delamanid, LIN – linezolid, S – susceptible, R – resistant, U – uncertain, nd – not determined.
